# How to integrate wet lab and bioinformatics procedures for wine DNA admixture analysis and compositional profiling: Case studies and perspectives

**DOI:** 10.1371/journal.pone.0211962

**Published:** 2019-02-12

**Authors:** Rita Vignani, Pietro Liò, Monica Scali

**Affiliations:** 1 Department of Life Science, University of Siena, Siena, Italy; 2 Serge-genomics, Siena, Italy; 3 Computer Laboratory, University of Cambridge, Cambridge, United Kingdom; Consiglio per la Ricerca e la Sperimentazione in Agricoltura, ITALY

## Abstract

The varietal authentication of wines is fundamental for assessing wine quality, and it is part of its compositional profiling. The availability of historical, cultural and chemical composition information is extremely important for quality evaluation. DNA-based techniques are a powerful tool for proving the varietal composition of a wine. SSR-amplification of genomic residual *Vitis vinifera* DNA, namely Wine DNA Fingerprinting (WDF) is able to produce strong, analytical evidence concerning the monovarietal nature of a wine, and for blended wines by generating the probability of the presence/absence of a certain variety, all in association with a dedicated bioinformatics elaboration of genotypes associated with possible varietal candidates. Together with WDF we could exploit Bioinformatics techniques, due to the number of grape genomes grown. In this paper, the use of WDF and the development of a bioinformatics tool for allelic data validation, retrieved from the amplification of 7 to 10 SSRs markers in the *Vitis vinifera* genome, are reported. The wines were chosen based on increasing complexity; from monovarietal, experimental ones, to commercial monovarietals, to blended commercial wines. The results demonstrate that WDF, after calculation of different distance matrices and Neighbor-Joining input data, followed by Principal Component Analysis (PCA) can effectively describe the varietal nature of wines. In the unknown blended wines the WDF profiles were compared to possible varietal candidates (Merlot, Pinot Noir, Cabernet Sauvignon and Zinfandel), and the output graphs show the most probable varieties used in the blend as closeness to the tested wine. This pioneering work should be meant as to favor in perspective the multidisciplinary building-up of on-line databanks and bioinformatics toolkits on wine. The paper concludes with a discussion on an integrated decision support system based on bioinformatics, chemistry and cultural data to assess wine quality.

## Introduction

In the last few years the quality and safety of food products has become an essential requirement guaranteed to consumers in all fields of agricultural production. Regulations regarding wine production have profound effects on the character and quality of the wine. Such regulations can be found on local, national, and international levels, and regulatory laws must communicate with all the others [1].

In Europe, especially for some Countries such as France and Italy, wine production represents a significant part of gross domestic production (GDP). Regulatory efforts arose immediately after the terrible phylloxeric epidemic attack which promoted the spread of low-quality vines resulting from the hybridization of *V*. *vinifera* accessions with non *vinifera* spp, and the use of grapevines with high sugar content imported from Northern African regions. The European wine industry experienced significant growth during the 1960s economic expansion. As soon as the EEC began the process of unifying the wine industry, Regulation 24/62 defined a minimal requirement of wine quality in order to enter the international market. Following the EEC criteria for wine quality and in order to organize the many varieties of wine throughout Italy the Italian government established Law 930/1963 classifying wines with the title of DOC ("Denominazione di Origine Controllata"). This system of classification further evolved, and in the 1980s the first DOCG ("Denominazione di Origine Controllata e Garantita") emerged, a title awarded to any variety that had maintained DOC status for at least five years.. Today, in order to commercialize a wine as monovarietal, with the exception of specific wines such as the Brunello di Montalcino and Barolo which contain 100% of a specific vine, it must contain at least 85% of the grape variety mentioned on the label.

Fraudulent use of regional names was especially widespread as producers saw that wines from specific regions demanded a significant premium even though the inputs were practically the same as their own wines.

Since 1983, and according to US regulation, in order to be labeled with a particular varietal name, a particular AVA (American Viticultural Areas), county, or state, a wine must contain at least 75% of the grape variety declared on the label.

The need to develop molecular analysis to confirm the authenticity of products has strongly increased after industry globalization in order to confirm food authenticity and to prevent fraudulent actions [2].

A variety of analytical techniques, characterized by a high level of specificity, can be applied to raw materials or semi-processed products. [3]. Further, DNA is well known to be useful for individual grapevine genotype identification studies through PCR [4], and for other species even in quite extreme chemical and physical conditions, such as those encountered in forensic cases [5], paleobotany [6, 7], paleontology [8] and in cases of ancient DNA [9]. Varietal assessment methodologies can be based on proteins, metabolites or DNA analysis for food authentication. While protein or proteomic-based approaches (immunological or electrophoretic assays) and metabolite content detection (HPLC, or NMR) are limited by uncontrolled effects due to environmental conditions and industrial procedures [10, 11] the DNA-based methodologies, even if limited by DNA degradation, are slightly independent from environmental conditions. Food and beverages composition may thus be analyzed for their varieties/breed composition by DNA testing [2] to prevent adulteration. In fact, although DNA is denatured, or degraded, by heat like proteins it can be still detected through the *polymerase chain reaction* (PCR) [12]. Although PCR is widespread in the field of food testing it raises problems and difficulties, especially when heterogeneous matrixes or processed food are analyzed [13]. The DNA extracted from food products tends to be low in quantity and to be highly degraded in relation to the extent to which the food has been processed. Generally speaking, exposure to heat results in the fragmentation of high molecular weight DNA, and likewise physical and chemical treatments cause random breaks in the strands reducing fragment size [14]. However, DNA degradation doesn’t seem to impair the use of a molecular authentication approach [15, 16]. The existence and state of DNA in wine after the complex technological procedures it undergoes; such as decanting, filtration, metabolic digestion due to fermentation, aging and autolysis, remains a debated and controversial issue [17, 18]. Data on the structural and functional characterization of grape genes accumulate exponentially, also thanks to the bioinformatics applications to genomic data interpretation finalized in the decoding of grapevine gene function. A large number of structural variants and SNPs were identified in the table grape ‘Sultanina’ and ‘Nebbiolo’, constituting a novel and powerful tool for genomic studies to support marker-assisted breeding in grapevines. [19, 20, 21]. To achieve optimal knowledge of grapevine physiology, international research has explored the potential of many different scientific disciplines in the last 10 years, including functional analysis, genotyping and phenotyping by trait ontology, and bioinformatics (IGGP program).

**Fig 1:**
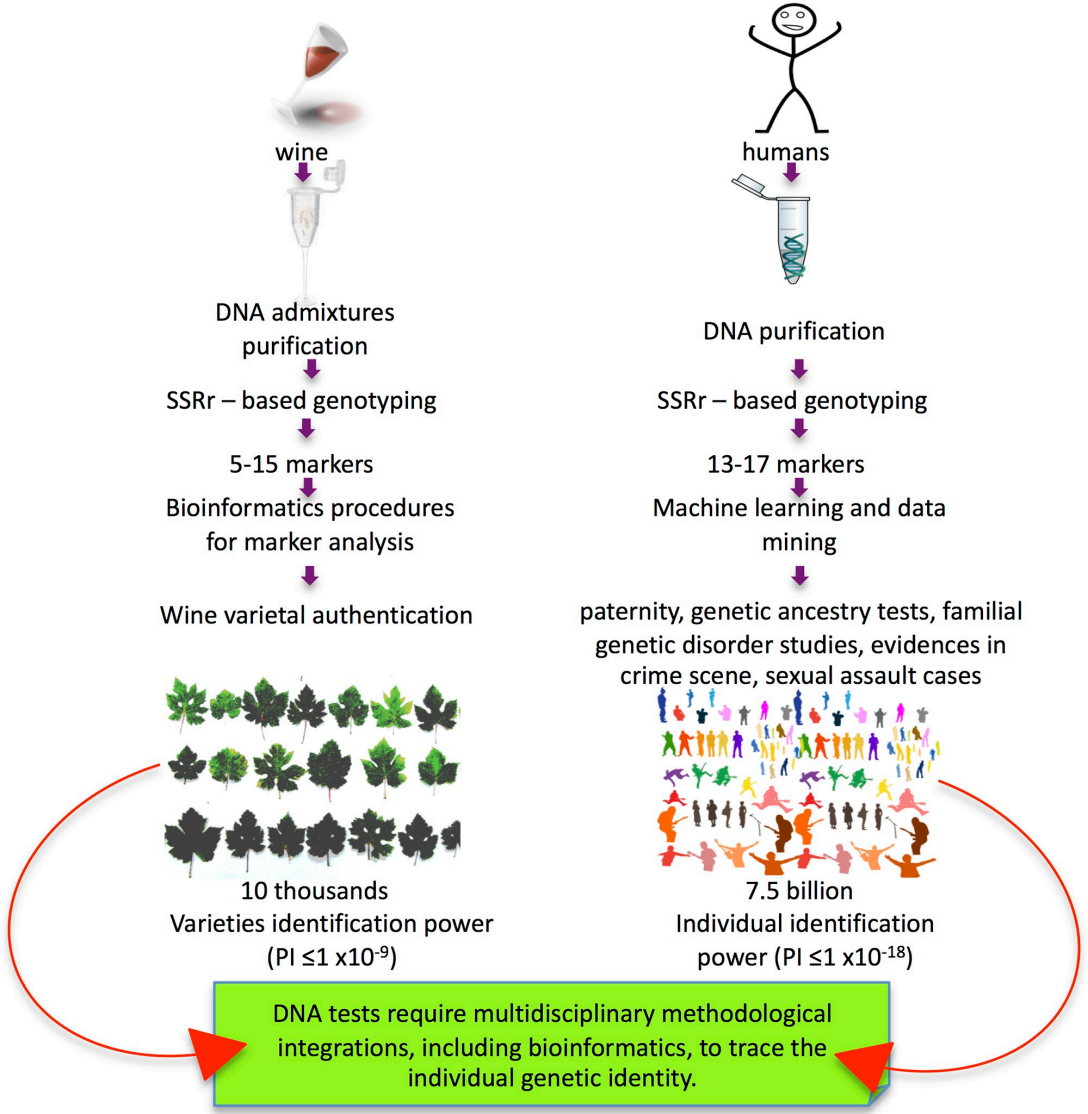
Methodology similarities between WDF and forensic DNA-testing. Methodological similarities between WDF and human genotyping both require DNA purification, and in forensic applications, DNA admixture analysis. The individual genotype is generally reconstructed by nuclear genomic DNA amplification targeting multiple SSR loci. The identification power of the test is designed to detect genotype rarity in billions of individuals (human) or in thousands (grapevine varieties). WDF test conditions can be less stringent than those carried out for humans due to smaller population size and the less complicated population structure, principally determined by the clonal propagation of the grapevine. Bioinformatic data elaboration from wine is an essential step for WDF’s validation.

Wine varietal content is an essential feature for proving wine correspondence to productive regulatory dispositions, for guaranteeing authenticity of the beverage in the consumer’s eyes, and to defend wine makers and their valuable products on the market against counterfeiting attempts. Since wine consists of a complex mixture of molecules, including substances negatively interfering with DNA extraction procedures, it is not feasible to expect good quality DNA. It is thus still essential to have optimal procedures for wine DNA extraction that can produce DNA fractions compatible with consistent and reliable *Vitis vinifera* genome target amplification. However, even given the historical [22, 23, 24, 25] and more recently proven reliability of the molecular approach for varietal authentication [26, 27, 28, 29, 30, 31, 32], the efficacy of wine DNA extraction and genotyping should be further tested on several wine types, and a statistical supporting tool for allele observation in Wine DNA Fingerprinting (WDF) and genotyping assignment must still be developed. Several analytical methods for wine varietal authentication have been used. Among others, Fourier Transform Infra Red (FTIR) spectroscopic analysis of wines [33, 34], High Pressure Liquid Chromatographic (HPLC) analysis of anthocyanins and shikimic acid in wines [35], NMR profiling [36], and Direct mass spectrometry analyses of the volatile content [37, 38]. Each of these approaches presents reliability challenges in determining varietal authenticity, and the general tendency is to integrate different analytical approaches to determine the varietal nature of wine [39]. The anthocyanin and shikimic acid-based method has provided limited success in authenticating only specific, pure varietal wines. However, according to US regulation wines may still be identified as varietal wines as long as they contain at least 75% of the grapevine variety reported on the label, and this particular method has significant limitations in authenticating blended wines. An additional challenge of this method is the chemical property of polyphenolics (anthocyanins) in wine. It is known that anthocyanin concentrations slowly decrease over time, and HPLC methods are successful only for red young wines [40]. Although chemical-based methods are continuously improving, we believe that at present the DNA-based wine varietal authentication based on SSR-genotyping (WDF) deserves consideration since it is relatively simple, methodologically very similar to human forensic genotyping, and relatively low cost (Fig 1). Historically, cultivar identification of wines using DNA biomarker technology was problematic due to the very low level of DNA fragments in processed wines. However, recent advances in the extraction protocol of DNA fragments and the utility of PCR technique have allowed researchers to overcome sensitivity issues in DNA analysis in wines.

It is important that analytical techniques be diffused so that small to medium sized labs located at the production and distribution level can practice quality assessment. This paper describes the use of WDF as well as the use of bioinformatics and statistical tools for varietal composition validation of different wines with increasing varietal complexity. The list includes experimental, monovarietal wines (Sangiovese-based), commercial monovarietals (Rosso and Brunello di Montalcino), wines produced in Northern Italy (Valpolicella, Verona), and unknown red and white blended wines widespread on the US market.

## Materials and methods

### Wines and grapevine references

The eighteen samples, either experimental or commercial wines used for bioinformatics elaboration of the genetic profiles, are listed in Table 1. The experimental Sangiovese wines (CB17 and IN7) obtained by small-scaling fermentation of the grapevine berries given at harvest (2014, 2015) by Case Basse (Montalcino, Italy) were kindly provided by the University of Florence. The commercial Italian wines were kindly provided by local wine makers (Azienda Palazzo e Caprili, Montalcino, Siena-Italy, Costa degli Ulivi-Verona, Italy, Mantellassi, Magliano in Toscana, Grosseto, Italy, Consorzio della Denominazione San Gimignano, San Gimignano Siena, Italy). The seven commercial varietal wines, four reds (947, 949, 950, 951) and three whites (953, 940, 948) were compared, respectively, to the following grapevines: Zinfandel, Merlot, Cabernet Sauvignon, Pinot Noir and the following grapevines: Riesling, Chardonnay and Sauvignon Blanc. All the grapevines used for the interpretation of the WDF profiles come from the Italian National collection held by Consiglio per la Ricerca in Agricoltura e l'Analisi dell'Economia Agraria, center for Enology and Viticulture (CREA, Conegliano Veneto, TV, Italy).

**Table 1:** Wines list. Wines listed from 1 to 18 with increasing varietal complexity either by nature (experimental to commercial), or detailed knowledge of their composition (grapevine variety, number of varieties used for the blend).

Wine name	Vintage	Year of analysis	Origin	Type (E = experimental) (C = commercial and certification, when applicable),	M = Monovarietal B = Blended varietal composition according to DOC or DOCG certification
**1. IN7**	2014	2015	University of Florence	E	M
**2. CB17**	2014	2015	University of Florence	E	M
**3. Brunello di Montalcino**	2014	2016	Montalcino, Siena, Italy	C, DOCG	M 100% Sangiovese
**4. Rosso di Montalcino**	2013	2016	Montalcino, Siena, Italy	C, DOCG	M 100% Sangiovese
**5. Alicante**	2010	2011	Magliano in Toscana, Grosseto, Italy	C, IGT	M 100% Alicante
**6. Cabernet Sauvignon, varietal**	2009	2012, 2016	USA	C	B
**7. Valpolicella Classico**	2015	2016	Fumane,Verona	C, DOC	B 45–95% Corvina or up to 50% Corvinone, 5–30% Rondinella
**8. Merlot, varietal**	2011	2012, 2016	USA	C	B
**9. Amarone**	2015	2016	Fumane,Verona	C, DOCG	B 45–95% Corvina or up to 50% Corvinone, 5–30% Rondinella
**10. Vernaccia di San Gimignano 1**	2010	2012	San Gimignano, Siena, Italy	C, DOCG	B 85% Vernaccia di San Gimignano 15% any grapevine allowed in Tuscany
**11. Vernaccia di San Gimignano 2**	2010	2012	San Gimignano, Siena, Italy		
**12. Unknown red varietal wine 947**	2014	2015, 2016	USA	C	B
**13. Unknown red varietal wine 949**	2014	2015, 2016	USA	C	B
**14. Unknown red varietal wine 950**	2014	2015, 2016	USA	C	B
**15. Unknown red varietal wine 951**	2014	2015, 2016	USA	C	B
**16. Unknown white varietal wine 940**	2014	2015, 2016	USA	C	B
**17. Unknown white varietal wine 948**	2014	2015, 2016	USA	C	B
**18. Unknown white varietal wine 953**	2014	2015, 2016	USA	C	B

### DNA purification from grapevine leaves

Plant DNA was extracted from grapevine leaf tissues using the commercial DNeasy Plant Mini Kit (QIAGEN, Germany), following manufacturer instructions. Homogenization of plant tissues was obtained by grinding 100 mg of fresh leaf with a plastic pestle in a 1.5 mL Eppendorf tube. Final DNA samples were diluted in 100 μl of Buffer AE (10 mM Tris-HCl; 0.5 mM EDTA; pH 8.0).

### DNA purification from wines

This protocol was described in [31] (The TECP method: Tris-EDTA-CTAB) and after updated in [41]. Before processing, the wine sample (approximately 300–400 ml) was precipitated at -80°C by adding 1 volume of 0.3M sodium acetate NaOAC (3M, pH 5.2) and 1 volume of isopropanol, and it was kept at least three days overnight.

After precipitation the samples were centrifuged at 8,000 rpm for 30 minutes at a temperature of +4°C in a JA-10 Beckman rotor. Pellets were resuspended in 20 ml of preheated TEX Buffer [1M hydroxymethylaminomethane-hydrogen chloride (Tris-HCl) pH 8.0; 1,4 M sodium chloride (NaCl); 20 mM ethylenediaminetetraacetic acid (EDTA); 3% (w/v) hexadecetyltrimethylammonium bromide (CTAB); 1% (v/v) β-mercaptoethanol], after homogenization by vortexing the samples were incubated for 1 hour at 65°C. At this point, the first organic solvent extraction was carried out by adding 1 volume of chloroform-isoamyl alcohol (24:1) (v/v). Tubes were vortexed and centrifuged at 10,000 rpm for 10 minutes at +4°C. The supernatant was recovered and transferred into another tube and 0,1 volume of CTAB (10%) was added to samples. The second organic solvent extraction took place by adding 1 volume of chloroform-octanol (24:1) (v/v), vortexed and centrifuged samples at 10,000 rpm for 10 minutes. At this point, 1 volume of 2-propanol was added at the supernatant recovered and samples were left to precipitate at -80°C overnight.

After precipitation samples were centrifuged at 8,000 rpm for 30 minutes, and the pellet was dissolved in 500 μl of TE Buffer (3M Tris-HCl, pH 8.0; 0,5M EDTA) and 40 μl of proteinase K (20mg/ml). Tubes were incubated for 30 minutes at 50°C. Then, 500 μl of phenol was added to samples which were vortexed for 1 minute and centrifuged at 14,000 rpm for 10 minutes. The supernatants were recovered and 1 volume of chloroform-isoamyl was added. Tubes were vortexed for 1 minute and centrifuged at 14,000 rpm for 7 minutes. The supernatants, containing nucleic acid, were precipitated with 2 volume cold 95% ethanol in presence of 2.5 M ammonium acetate for 30 minutes at -80°C. After precipitation the tubes were centrifuged for 30 minutes at a temperature of +4°C.

From this point, the extraction was completed following the Qiagen qiaprep spin mini Kit (Germany). DNA samples were eluted in 65 μl of Elution Buffer (10 mM Tris-HCl; 0.5 mM EDTA; pH 8.0) and they were stored at a temperature of -20°C.

### SSR-genotyping and allele dimensioning

Wine DNA Fingerprinting (WDF) was obtained by amplifying the nuclear *Vitis vinifera* DNA from plants and wine at the following SSRs loci: VVMD21, VVMD25, VVMD27, VVMD32, VVMD34, [42], VVS2 [43] VrZag 21, VrZag 79, VrZag 83 [44]. PCR reactions were carried out in at least three technical replicas for the wines and in double for the plants in an Eppendorf Mastercycler gradient PCR in a total volume of 12.5μl containing 2.5μl of genomic DNA, 0.25 mM dNTPs, 0.25μM each primer (one of them being fluorescein labeled), 1X Green GoTaq Reaction Buffer containing 1.5 mM MgCl2, 0.1U Go Taq DNA Polymerase (Promega). PCR conditions included an initial denaturation step of 5’ at 95°C, 39 cycles of 30” at 95°C, 30” at 48–56°C, 1’ at 72°C and a final extension of 10’ at 72°C. The PCR products were separated on 2% agarose gel stained with ethidium bromide to identify possible imperfections and to decrease the rate of failure in capillary electrophoresis. 1 μl of PCR product and 10 μl of a loading mix consisting of Highly deionized formamide (Sigma Aldrich) and 0.2 μl internal size standard (Et-Rox-400, HD-400 Life Technologies) were denatured at 95°C for 2’ and kept on ice. The polymorphism of SSR sites was examined by the capillary electrophoresis, based on laser scanning of fluorescence-marked DNA fragments. Genotyping was done on an ABI3130 DNA sequencer and fragment analyzer (Life Technologies). Fluorescently labelled amplified fragments were scored by GeneMarker free software. An estimate of the discrimination power of the test in resolving varieties was determined by calculating the probability of identity (PI) with the program IDENTITY v.4.0 [45] for each SSR marker set used as described in [46].

### Methods from bioinformatics, statistics and machine learning for wine admixture analysis, data interpretation and decision support system

There are various bioinformatics, statistical and machine learning methods to analyze molecular, biochemical and sensory wine data. We can first distinguish methodologies for classification with respect to Statistical Modeling for Degradation of wine properties. For the latter, we suggest that useful models could be inspired by recent literature on generalized cumulative damage approaches and hierarchical Bayesian of non linear change point analysis of degradation data [47]. For the former, a further distinction is between chemical analysis for which exists a rich machine learning and dataset framework (see for instance the UCL machine learning repository, https://archive.ics.uci.edu/ml/datasets/wine) and a statistical and machine learning-based bioinformatics.

There are statistical bioinformatics methods that are in use in biomarker analysis in various fields, such as forensic, NGS and phylogenetic analysis [48]. Here, while considering a not too technical user, we explored several methods and we arrive to the conclusion that both methods used in clustering and methods from phylogenetic analysis for discrete characters could be of utility. On a different line of thought we believe that the ultimate target is to integrate nucleic acids analysis with chemical and compositional analysis. Therefore, general statistical and machine learning methods that can be used for both types of data could provide effective integration. A broad list includes:

#### Clustering methods

This class of methods inputs vector data for calculating different distance matrices. Various methods are available, including hierarchical, k-means, shared-nearest neighbor and many others. For example, **Hierarchical clustering** aims to build a hierarchy of clusters. It takes as input a matrix representing pairwise distances between entities, joins the closest pairs of entities, then calculates a new distance between this merged entity and all others and repeats until all entities have been merged. **K-means clustering** aims to group data into a predefined number (**K**) of clusters by first randomly assigning entities to clusters, calculating a mean profile of each cluster, determining the inter-and intra-cluster distances, then assigning entities to the nearest cluster and recomputing the mean profiles. This process is repeated either in a predetermined number of times, or until the entities do not change their cluster membership. Interestingly, Dynamic clustering and Time-variant clustering could be used with longitudinal data i.e. they can be used to generate ‘branching’ patterns of sample clusters over time and be predictive of changes in the wine quality trajectory.—Here, we consider the capability of a small\medium-sized lab and we found PvClust providing effective solutions for the classification.

In order to measure the accuracy of the clustering approach and assess the uncertainty in the hierarchical cluster analysis carried out in this work, or each cluster in hierarchical clustering, we have calculated the p-values by using the multiscale bootstrap resampling, performed by the Pvclust package in R. In particular, "pvclust" allows calculating two types of p-values, the approximately unbiased (AU) p-value and the bootstrap probability (BP) value. As described in [49], by randomly sampling elements of the data it is possible to generate thousands of bootstrap samples, so that bootstrap replicates of the dendrogram are obtained by repeatedly applying the cluster analysis to them. The bootstrap probability (BP) value of a cluster is the frequency that the cluster appears in the bootstrap replicates, and represents a biased probability. Conversely, the AU p-value, which is computed by the multiscale bootstrap sampling, represents a better approximation to unbiased p-value, if compared to the BP value computed by normal bootstrap resampling. Thus, pvclust performs hierarchical cluster analysis via function hclust and automatically computes p-values for all clusters contained in the clustering of original data. It also provides graphical tools such as the plot function or the "pvrect" function, which highlights clusters with relatively high/low p-values.

#### Dimensionality reduction techniques

This class includes principle (PCA) and independent (ICA) component analysis, Multidimensional scaling (MDS), t-distributed stochastic neighbour embedding (t-SNE). A simple effective methodology, implemented in several softwares, is the Principal component analysis (PCA): the method finds axes or directions that are linear combinations of variables that maximize the total variation in the data set and are orthogonal to each other. The key step is the visualization of a small subset of dimensions that capture the important information in the data structure. Note that the availability of a large amount of data could allow the use of more advanced methods such as autoencoders neural networks which could be described as non linear PCA. Here, we consider the capability of a small\medium-sized lab we applied PCA (with the R tool “ggbiplot”) to investigate which experiments contributed the greatest to differentiating considered wines.

#### Phylogenetic techniques

Phylogenetic methods have been developed for a variety of data, including binary, discrete, continuous (see for instance the impressive repository at the URL: http://evolution.gs.washington.edu/phylip/software.html). We found a clustering method, commonly used in phylogenetic bioinformatics, particularly useful: Neighbor joining is a bottom-up, agglomerative clustering method widely used in phylogenetics and created by Naruya Saitou and Masatoshi Nei [50]. The method uses a distance matrix that specifies the distance between each pair of samples from different experiments based on the co-occurrence of markers. The algorithm starts with a start tree i.e. a completely unresolved tree and iterates over the following steps until the tree is completely resolved and all branch lengths are known. The important distinction with respect to Bioinformatics analysis of DNA sequences is the use of a threshold to discriminate wines that do not have markers in common.

## Results

### DNA extraction from wines

The quality of total DNA extracted from wines was evaluated on the basis of SSR amplification efficiency so as to have at least 7 nuclear markers to be correctly amplified. The number of markers can be slightly lowered in monovarietal wines (S1 Table), due to the straightforwardness of the comparison between the wine and plant profiles. However, total DNA and VvDNA quantity and quality obtained from each wine from an early temporal phase of this study are reported as supplementary material in S2 Table. The limiting qualitative features of the wine DNA molecule seem not to impair the readability of the grapevine template DNA in PCR-downstream applications, as demonstrated by the SSR profiles obtained (S1 Fig). The input data files, consisting of each validated allele observed both in plants and in the wine (S3 and S4 Tables), were used to elaborate a graphical output of data through bioinformatics elaboration.

### Statistical analysis inspired by bioinformatics on wine DNA fingerprints

The algorithm used to construct genetic distance trees, specific for the analysis of microsatellite loci, takes into account not only the distance between two individuals but also the distance between an individual and all the others, thus reconstructing a distance report.

**Fig 2:**
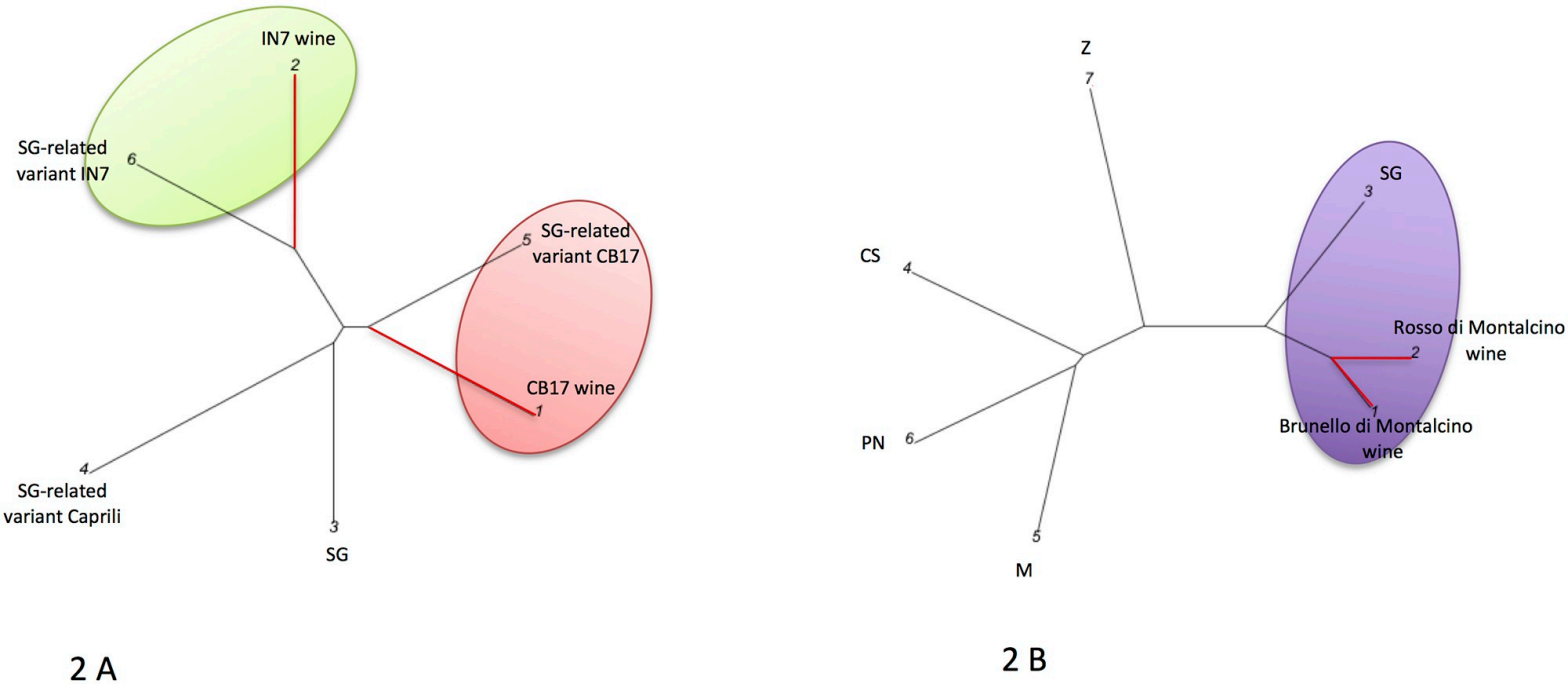
Graphical representation of the Sangiovese-based WDFs. Sangiovese Wines DNA fingerprints (WDF) were used for constructing graphical outputs of the genetic distances among wines and grapevines genotypes. **2 A**: wines: 1 = small scale fermented CB17; 2 = small scale fermented IN7. Grapevines: 3 = Sangiovese (SG); 4 = SG-related variant Caprili; 5 = SG-related variant CB17; 6 = SG-related variant IN7. **2 B**: wines: 1 = Brunello di Montalcino; 2 = Rosso di Montalcino. Grapevines: 3 = Sangiovese (SG); 4 = Cabernet Sauvignon (CS); 5 = Merlot (M); 6 = Pinot Noir (PN); 7 = Zinfandel (Z). Red branches link the wines, black ones the grapevines. Only the wines are shown with explicit names; the numbers associated to a two-capital letter acronym code refer to the reference grapevines.

**Fig 3:**
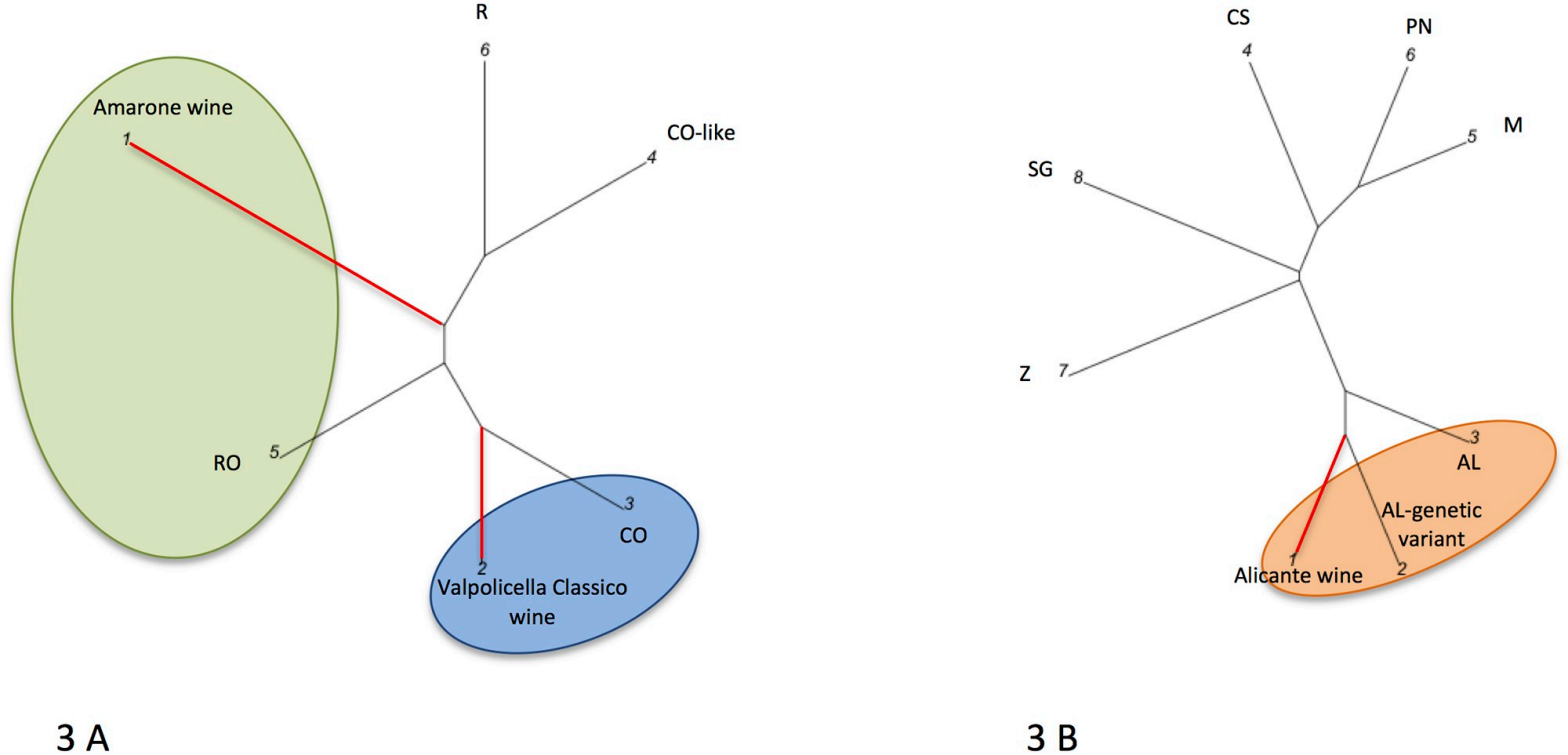
Graphical representation of the Valpolicella region (Veneto, Italy) and an IGT wine from Tuscany. Amarone, Valpolicella Classico (Valpolicella, Veneto, Italy) and Alicante wine (Tuscany, Italy) DNA fingerprints (WDFs) graphical outputs. **3 A**: wines: 1 = Amarone; 2 = Valpolicella classico. Grapevines: 3 = Corvina (CO); 4 = Corvina-like (CO-like); 5 = Rondinella (RO); 6 = Riesling (R). **3 B**: 1 = Alicante wine. Grapevines: 2 = Alicante genetic variant (AL-genetic variant); 3 = Alicante (AL); 4 = Cabernet Sauvignon (CS); 5 = Merlot (M); 6 = Pinot Noir (PN); 7 = Zinfandel (Z); 8 = Sangiovese (SG). Red branches link the wines, black ones the grapevines.

**Fig 4:**
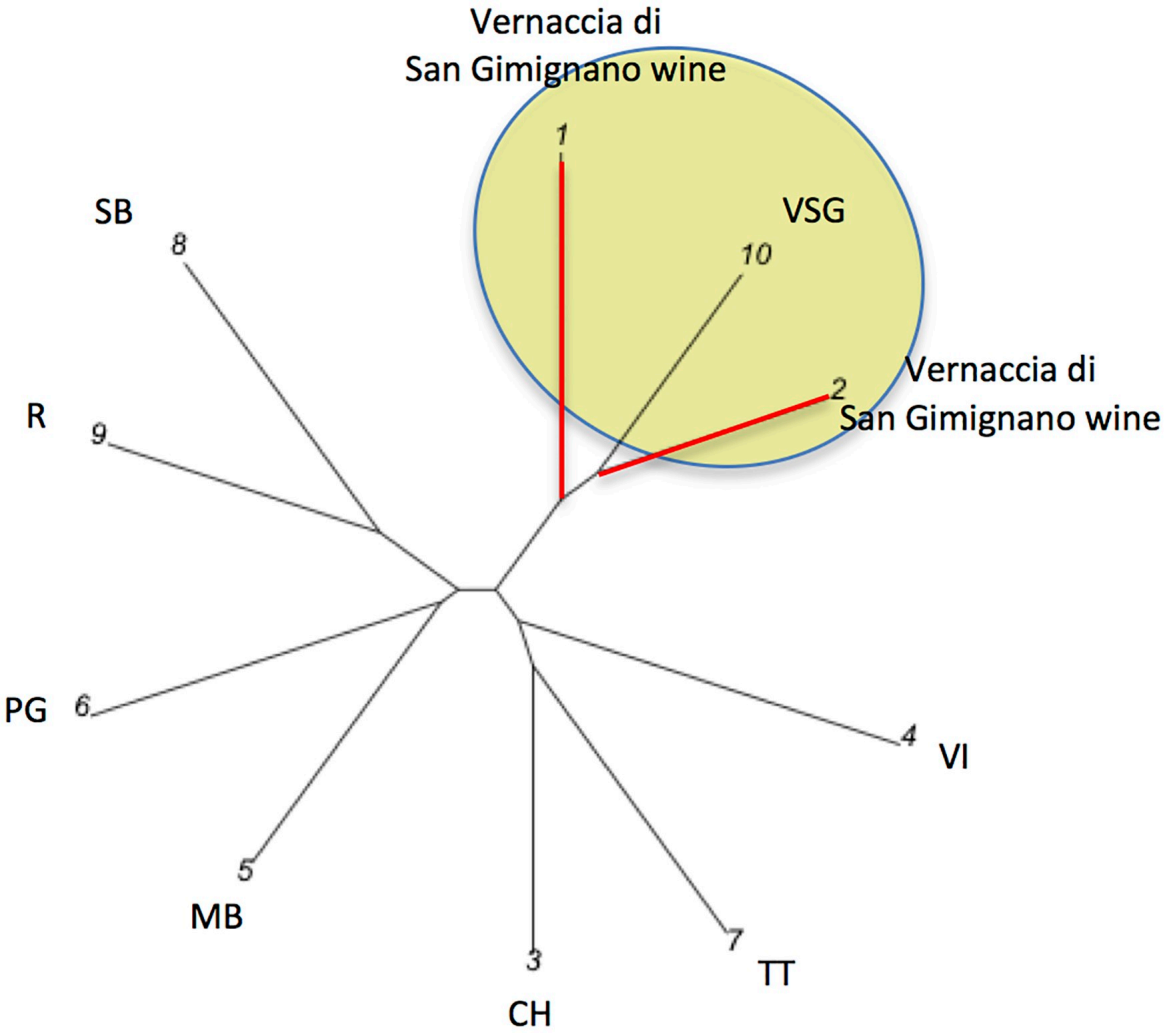
Graphical representation of the Vernaccia di San Gimignano wines WDF. Vernaccia di San Gimignano wines are closer to the Vernaccia di San Gimignano major varietal component. Wines: 1 and 2 = Vernaccia di San Gimignano. Grapevines: 3 = Chardonnay (CH); 4 = Viognier (VI); 5 = Manzoni Bianco (MB); 6 = Pinot Gris (PG); 7 = Trebbiano Toscano (TT); 8 = Sauvignon Blanc (SB); 9 = Riesling (R); 10 = Vernaccia di San Gimignano (VSG). Red branches link the wines, black ones the grapevines.

**Fig 5:**
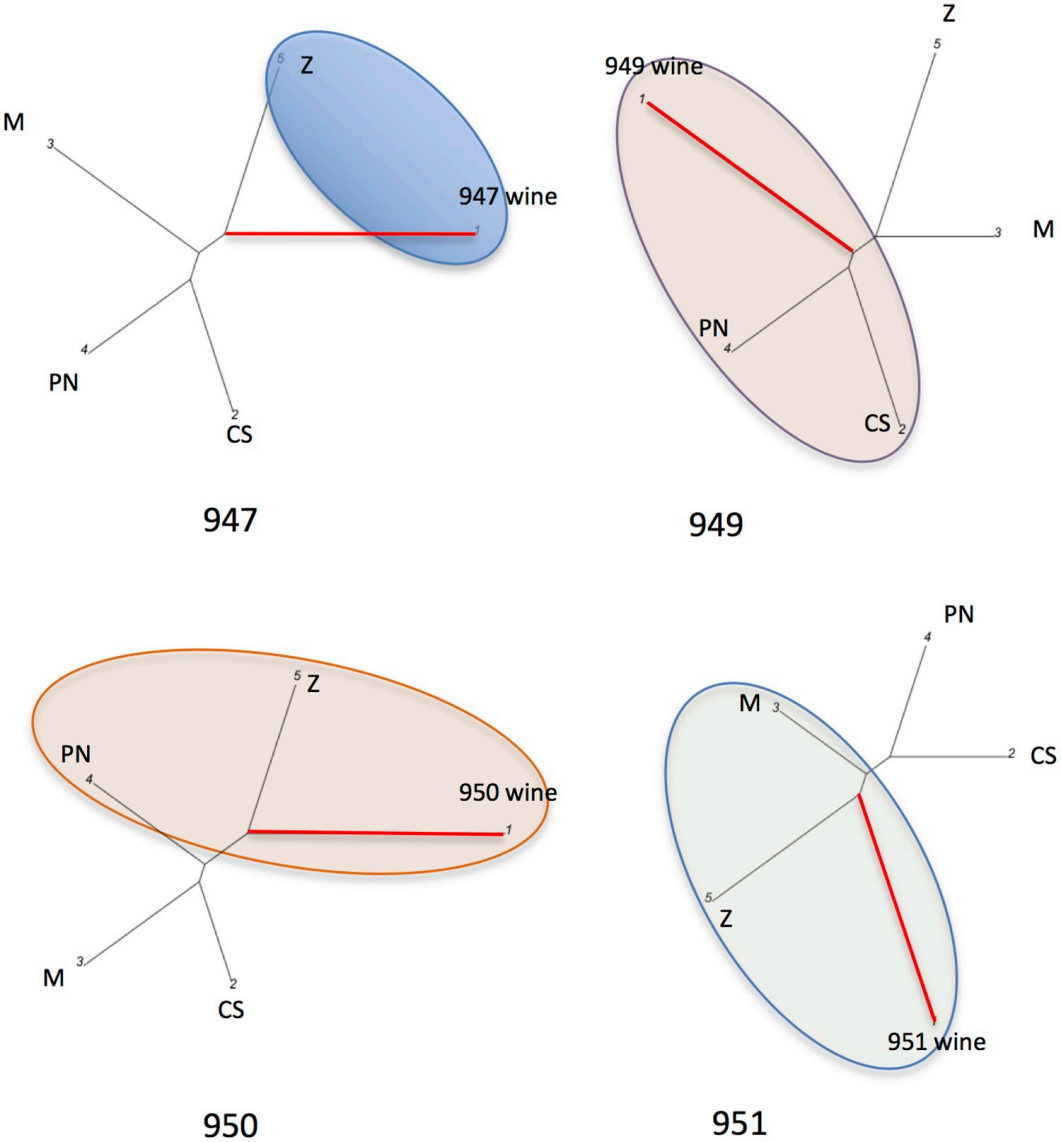
Graphical representation of the WDFs of four varietals, blended, unknown wines from the US market. Red wines 947, 949, 950, 951 from the American market were subjected to WDF and subsequent bioinformatics elaboration of genetic profiles. In all graphs 1 is the wine, the grapevines are: 2 = Cabernet Sauvignon (CS); 3 = Merlot (M); 4 = Pinot Noir (PN); 5 = Zinfandel (Z). 947 was revealed to be a varietal Zinfandel, 949 a varietal Cabernet Sauvignon, 950 Pinot Noir, 951 a Merlot. Secondary, undeclared varietal components might be present in the blend, such as Zinfandel in the 950 and 951, Pinot Noir in the 949. Red branches link the wines, black ones the grapevines.

In Fig 2 the experimental and commercial Sangiovese WDFs are compared to possible candidate grapevine components. In Fig 2(A) the small scale fermented CB17 and IN7 experimental wines (1 and 2) were compared with Sangiovese-related variant CB17 (5), Sangiovese-related variant “Caprili” (4), Sangiovese-related variant IN7 (6) and Sangiovese (3). In Fig 2(B) the Brunello and Rosso di Montalcino wines (1 and 2, respectively) were compared with plant references: Sangiovese (3) Zinfandel (7) Cabernet Sauvignon (4), Pinot Noir (6) and Merlot (5). Red branches link the wines, black ones the grapevines. In particular, the small-scale fermented wines "IN7 and CB17" produced using selected berries obtained from pure genetic variants of Sangiovese cluster with IN7 and CB17 Sangiovese variants, respectively, and not to the standard Sangiovese. The commercial monovarietal wines Brunello and Rosso di Montalcino are closest to the Sangiovese and appear clearly separated from the Zinfandel, Cabernet Sauvignon, Pinot Noir and Merlot. Fig 3 depicts the graphical representation of the WDFs of wines from Veneto (Valpolicella, Italy) and an IGT wine from Tuscany. In Fig 3A the following are reported in order: the wines linked with red branches Amarone (1) and Valpolicella classico (2); the grapevines Corvina (3), Corvina-like (4), Rondinella (5) and Riesling (6). 3 B: Alicante wine (1); the grapevines Alicante genetic variant (2), Alicante (3), Cabernet Sauvignon (4), Merlot (5), Pinot Noir (6), Zinfandel (7), Sangiovese (8). In Fig 3A the Amarone and Valpolicella Classico wines are closest to the Rondinella and Corvina, respectively, than to any other plant reference. The Alicante monovarietal wine (Fig 3B) reflects the presence of genetic variants in the vineyard, being closest to the Alicante genetic variant (2) and Alicante (3), than to Cabernet Sauvignon, Merlot, Pinot Noir, and Zinfandel. Both Vernaccia di San Gimignano white blended wines (1, 2) (Fig 4) are closer to the Vernaccia di San Gimignano due to the presence of this grapevine variety as main component (10), than to grapevine profiles of: Sauvignon Blanc (8), Riesling (9), Pinot Gris (6), Manzoni Bianco (5), Chardonnay (3), Trebbiano Toscano (7), and Viognier (4), which branch out from the wine profiles. The four blended red wines taken from the American market and delivered to us as unknown samples, were tested for their WDF comparing the wine molecular profile with Cabernet Sauvignon, Merlot, Pinot Noir and Zinfandel grapevines (Fig 5). Each wine was closest to its main varietal component, 947 is a varietal Zinfandel wine, 949 is a varietal Cabernet Sauvignon, 951 a varietal Merlot wine and 950 a Pinot Noir varietal wine. The bioinformatics elaboration of the raw dataset is compatible with the hypothesis of secondary varietal components in the blend. The Zinfandel could be present in wines 950 and 951, while the Pinot Noir in the blend of wine 949.

**Fig 6:**
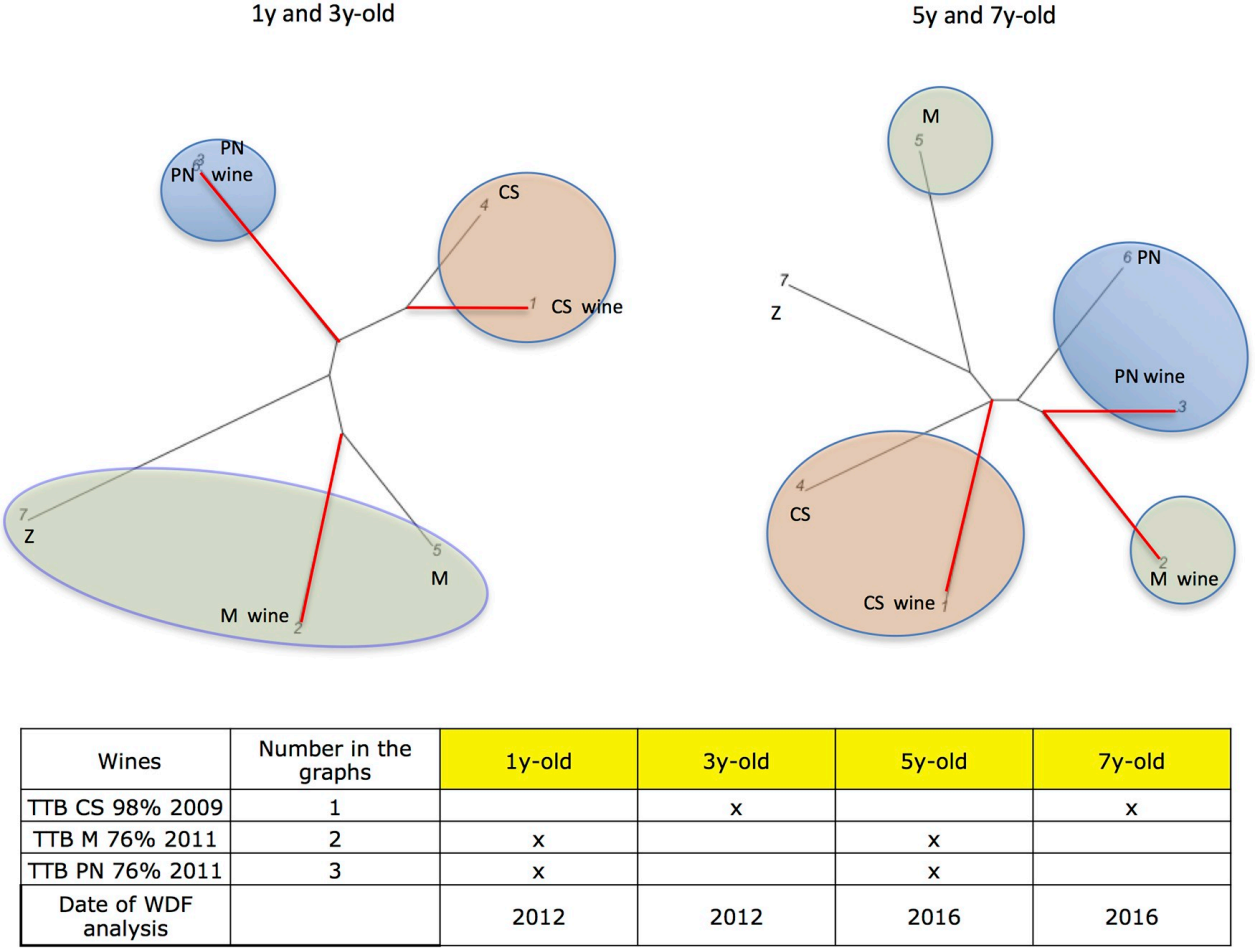
Consistency of WDF testing over time. Three varietal wines were tested for WDFs after one, three, five and seven years, since wine production. The varietal Cabernet Sauvignon wine (1 = CS wine) is grouped with Cabernet Sauvignon grapevine (4 = CS, light pink bubbles), after three, and seven years. The Pinot Noir varietal wine (3 = PN wine) is correctly grouped with Pinot Noir grapevine (6 = PN, blue bubbles), after one and five years. The Merlot wine (2 = M wine) is genetically related to the Merlot grapevine variety (5 = M, pale green bubbles), in the WDF performed after one year, while it loses the correct closeness to the main varietal component after five years. The Zinfandel (7 = Z) added to the Merlot wine at 1.238% appears to be detectable only after one-year. Red branches link the wines, black ones the grapevines.

The comparison of wine and grapevine genetic profiles might be done with the intent of responding to specific needs, including product compliance to specific National and International production and current regulations, and for addressing more general marketing needs targeting increasingly quality-conscious consumers in choosing food and wines. The WDF test is therefore universal since in principle any grapevine genotype might be compared to any wine. In order to check the consistency of the WDF over time three wines with known composition, Cabernet Sauvignon, Merlot, and Pinot Noir (Fig 6), were tested under identical methodological conditions at different wine ages. In particular, the 98% Cabernet Sauvignon (1) was analyzed after three and seven years respectively, while Merlot 76% (2), Pinot Noir 76% (3) were tested, after one and five years from production. Results demonstrate that the wine Cabernet Sauvignon varietal nature is still recognizable after 7 years. The Pinot Noir is recognizable after 5 years, while the Merlot loses the closeness to the Merlot grapevine profile after 5 years.

Bioinformatics can give us a graphical test of the assumptions made with the Random Match Probability (RPM) analysis between the WDFs and grapevines. The graphical representation of the WDF demonstrates how it is possible to estimate the predominant variety in a blended wine, and how it is possible to use the WDF and its graphical outputs for an immediate verification of the wine with its varietal nature declared in the label.

### Towards an integrated informative wine profiling databank system

**Fig 7:**
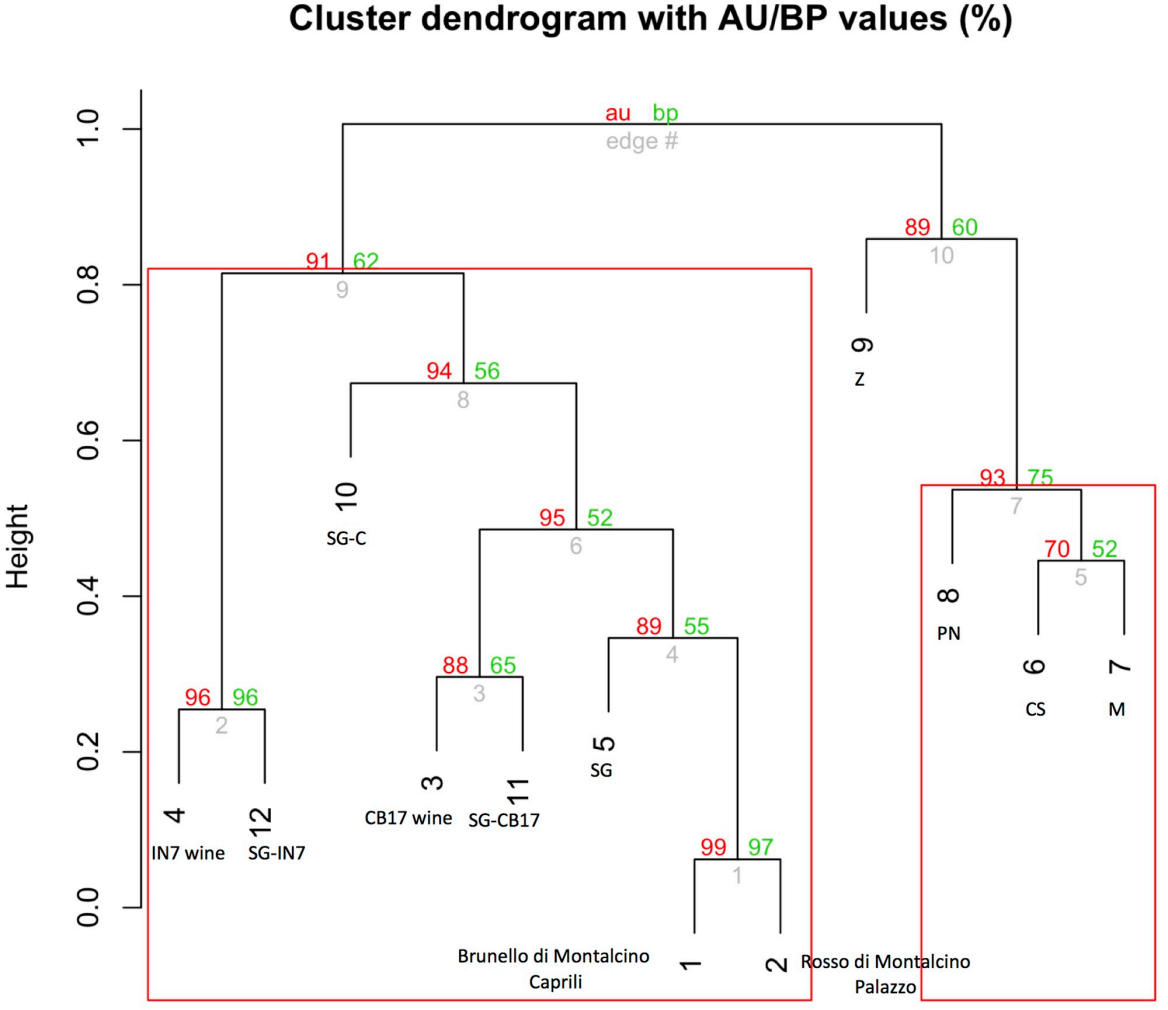
Clustering of Sangiovese-based wines. After data merging from multiple WDF experiments into a single dataset, the clustering reveals how the Sangiovese-based wines and experimental wines remain close to their varietal origin. Wines: 1 = Brunello di Montalcino Caprili 2014; 2 = Rosso Di Montalcino, Loia 2013; 3 = Small-scale fermented wine CB17; 4 = Small-scale fermented wine IN7. Grapevines: 5 = Sangiovese (SG); 6 = Cabernet Sauvignon (CS); 7 = Merlot (M); 8 = Pinot Nero (PN); 9 = Zinfandel (Z);10 = Sangiovese-related variant Caprili (SG-C); 11 = Sangiovese-related variant Case Basse CB17 (SG-CB17);12 = Sangiovese-related variant Case Basse IN7 (SG-IN7).

**Fig 8:**
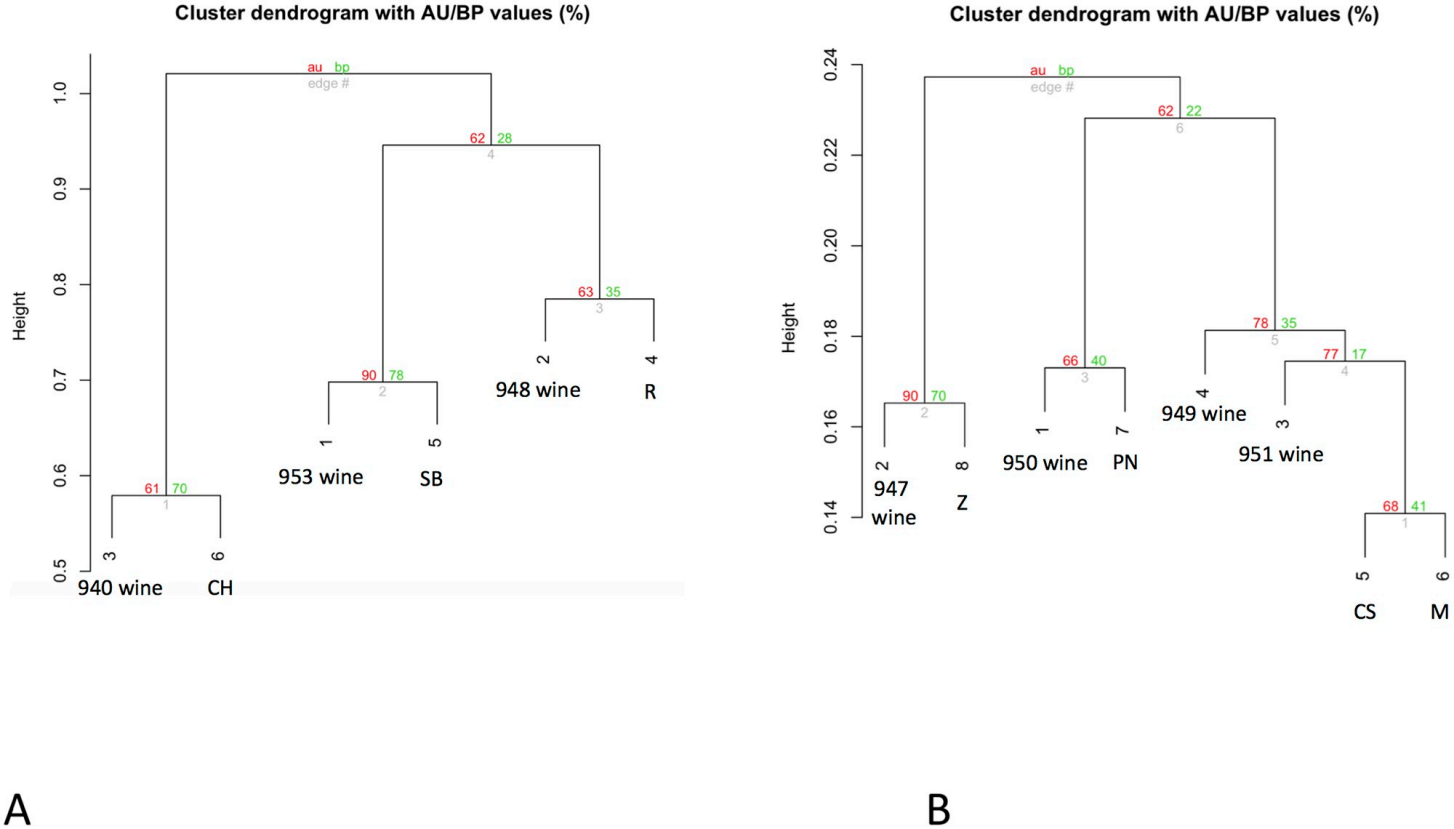
Clustering of US wines. After data merging from multiple WDF experiments into a single dataset, the clustering of the commercial US wines unambiguously resolves the varietal nature of the wines. **8 A**: White varietal wines (from left): 940 = 3, 953 = 1, 948 = 2, cluster with their respective varietal main components: 6 = Chardonnay (CH), 5 = Sauvignon Blanc (SB), and 4 = Riesling (R). **8 B**: Red varietal wines (from left): 947 = 2, 950 = 1, 949 = 4, 951 = 3 cluster with their respective varietal main components: 8 = Zinfandel (Z), 7 = Pinot Noir (PN), 5 = Cabernet Sauvignon (CS) and 6 = Merlot (M). The wines 949 (4) and 951 (3) and their respective original varieties Cabernet Sauvignon and Merlot, are put in the same branch due the high similarity between these two varieties at the molecular marker panel tested.

Multiple data was merged in single files in order to check the consistency and reliability of genetic data analysis in the optic of creating an integrated, informative system organized in on-line databanks for wine profiling that includes genetic, metabolomic and historical descriptions of the wines. In detail, three different data sets were used for subsequent bioinformatics elaboration. The merging action was done taking into account the geographical origin of wines: data set 1: Tuscan wines; dataset 2 (white blended wines 940, 958 and 953) and 3 (red blended wines 950, 951, 947, 949) from the US market, respectively. The discrimination power of each dataset after the merging of data was calculated with the Identity software to be: PI = 1,14 x 10^−3^, PI = 2,23 x 10^−7^ and PI = 9,31 x 10−^13^, for dataset 1, 2, 3, respectively. Fig 7 reveals that the WDF for the Sangiovese wines is consistent even after data merging, keeping the closeness of the monovarietal wines with the Sangiovese. Fig 8 shows the clustering of US wines (8A: whites; 8B: reds), where the varietal nature of each wine is depicted in the graph by the closeness to the respective grapevine variety.

## Discussion

### WDF technique: Advantages and drawbacks

Point-of-care technology demand along the wine making chain (in the food industry in general) focuses on rapid, low-cost and reliable analytical methodologies. In a mid-sized private laboratory setting, monitoring nonconformance is essential in determining areas where further improvements can be made. The DNA-based technologies are rapidly moving towards the use of portable biosensor-based technologies, which allow quick and low-cost approaches for wine varietal characterization [51]. In the near future the knowledge deriving from the rapid expansion of the genomic era will likely produce new biotechnological resources for food quality assessment and wine varietal authentication. A major breakthrough in grapevine genomics was achieved in 2007 with the sequencing of the *Vitis vinifera* cv. PN40024 genome, which was followed by an international effort to share the large amount of data arising from merging genomic knowledge of the genus *Vitis* [19].

Data from this paper demonstrate the reliability of the molecular approach for varietal authentication, the efficacy of wine DNA extraction and genotyping that were applied to several wine types with a common statistical validation strategy supporting the analysis. Accordingly, wines with increasing compositional complexity and age were chosen. Statistical validation tools developed on purpose for wine (see forward in this paper) renders easier the interpretation of wine varietal composition through the production of graphical outputs. The present work was primarily aimed at understanding the applicability and possible limiting factors of the varietal determination of wine by DNA analysis. Secondarily, a major goal has been the development of a strategy of WDF data validation. In commercial monovarietal wines the results confirm the reliability of the WDF, as previously demonstrated from earlier work [31]. In commercial blended wines which are the majority of the wines in the market the data obtained highlighted the need of sticking to a precautionary behavior, when predicting the varietal nature of a wine by DNA analysis. As a matter of fact, the study was conducted as a "*blind*" test on unknown, commercially available, wines taken from the US market, testing each sample against four different possibilities at time for the red wines (Merlot, Cabernet Sauvignon, Pinot Noir, Zinfandel) and against three different possibilities for the whites (Riesling, Chardonnay, Sauvignon Blanc). In synthesis, the work done lead to the following methodological critical points *i*) the choice of a SSR marker panel suitable for being efficient on grapevine DNA from wine and that was demonstrated to be at less risk of producing misleading genetic information (Allele drop-out and Allele drop-in); *ii*) the individuation of a strategy for electropherograms interpretation (use of technical replicas of wine and plant DNAs, these latter used as internal calibrators); *iii*) the setting of predictive analytical parameters and threshold values associated to each SSR panel (PI values) helpful in the WDF experimental planning; *iv*) development of statistical elaboration procedures, possibly usable for wine ontology databanks inspired by bioinformatics.

The SNP-on chip technology has never been used for wine varietal authentication, yet, likely due to major limiting factors especially the development of minimal set of functional/informative markers for varietal identification and grapevine DNA minimal quantity required. Compared to other molecular methods the WDF based on SSR typing has the advantage of the universality trait and the relatively low number of tests to be carried out in order to reach the reliability range of varietal identification (10K SNP chip versus 7–15 SSR markers) [52]. Since grapevine DNA from wine is likely degraded in most of the times, it appears to be much harder at least with actual DNA purification techniques to reconstruct grapevine varietal genotype based on targeting thousands of sites instead of dozens.

Main limitations of the actual WDF methodology consist primarily in the low-efficiency of wine DNA extraction techniques with respect to other high-through put analytical procedures, the rigor required for standardizing the analysis and a possible lack of comprehensive wine molecular databases.

### DNA stability in wine

From the molecular biology point of view wine is a difficult system to deal with due the complexity of the chemical environment and the great variability in terms of biological origin, technological processes and ageing which characterize the many kinds of wine worldwide. Most of the chemistry of wine that has been deeply studied correlates with wine fermentation [53, 54] and /or wine organoleptic quality traits [55, 56] Over the past years, nucleic acids, and DNA in particular, have been subjected to purification from wines; principally for the characterization of fermenting populations: yeasts and bacteria. Since 2000 scientific interest has been put into developing DNA extraction protocols from wine for wine varietal authentication purposes until recently [29, 30, 31, 32]. In general, when DNA degrades it tends to fragment into progressively smaller segments. A number of mechanisms have been postulated to account for this effect including hydrolytic cleavage, chemical oxidation and enzymatic degradation. In aqueous environments a low pH (3.0–4.2) and high ethanol content presumably inhibits the restriction nucleases activities that have characterized the matrix transformation from grapevine to wine. In other words, it is reasonable to expect that the microbial and yeast enzymes will chop most of the grapevine DNA during fermentation [27] within a short time. The persistence of DNA as free molecule in an aqueous environment was investigated in freshwater ecosystems [57], blood [58] under different conservation conditions, in fossils [59] and forensic cases [60]. Like the free-cell DNA in blood, which cancer cells continuously produce and release in the blood of cancer patients, all the case studies listed involve the assessment of critical quantities of DNA and imply DNA testing through an amplification of steps such as PCR or Next Generation Sequencing, associated to applications such as the massive parallel sequencing.

### Wine DNA quantification issue

Since roughly 2000 there has been an increasing interest in developing molecular diagnostic tools for wine varietal characterization primarily based on the use of molecular markers. Discussing how to determine grapevine DNA from wine could be tricky due to limited quantity (generally total DNA, including DNA deriving from yeasts and bacteria is below 30–40 ng/μL in aged wines, more than 5 years), and renders the spectrophotometry and florometry-based methods often unreliable. A more accurate result can be obtained by real-time PCR using specific probes [61]. In addition, artificial internal calibration probes can be used in real-time PCR as indexes for the presence of PCR inhibitors in the DNA extractions. Unpublished evidence [62] reveals that total DNA extracted out of one-year-old wines is comparable to that extracted from young grapevine leaves. In addition, wine DNA is a mixture deriving from the grapevine and fermenting microbiota, while leaf DNA derives from the nuclear and plastidial organelles. Thus, both the wine and the leaf contain DNA of multiple biological origins which can be selectively targeted by PCR depending on the marker type used.

SSR-based genotyping is a self-regulated test, in the sense that if the allelic sizes are validated against a robust data bank from wines to plants in each run, it is not strictly necessary to perform a nuclear *V*. *vinifera* quantification. Furthermore, each SSR marker has an intrinsic optimal range of target DNA quantity to maximize the probability of amplification success. According to forensics medicine, getting closer to threshold minimal quantity of 12.5 pg of DNA target below which stochastic effects occur it is recommended to perform at least triplicate amplification based consensus to allow for maximal data recovery [63]. The reliability of the test can be derived from the genotyping success rate among the different technical replicas of the same sample DNA.

### Capillary electrophoresis troubleshooting for allele sizing validation

Allele sizing and subsequent validation of the observed genotypes can be complicated by electrophoretic technical issues. Analyzing raw data is important for evaluating the result of the experiment: signal/noise ratio, amplitude and signal progression, correct response of the reaction and stroke in the capillaries, or spike signal abnormalities. The first fundamental step is to evaluate the presence of the signal in the data collected in the technical replicas of the same wine, and varietal references during the migration of the fragments which are fluorescently labeled and thus can be detected by the sequencer’s CCD camera. Occasionally the signal may be absent, or sporadic anomalous peaks can be monitored. When the amount of DNA is low, it may happen that the primers do not bind to the DNA target during the primer-annealing phase but match each other, resulting in primer-dimers formation. If the DNA fragments are not loaded into the capillaries, there is a lack of signal that is recorded only as background noise because the detector has not recorded any light emission. In that case, the software fails to recognize any data. This may be due to an instrument failure, lack of amplification reaction or low DNA concentration in the mixture. Low DNA concentration or the presence of contaminants such as proteins or DNA residues, may still give a very noisy signal making it difficult for the software to catch up. Instead, a large amount of DNA and primers in the reactions, such as in the plant varietal references, generates numerous fragments, which in turn gives rise to a very high signal but still interpretable by the software for the presence of an internal standard. However, an excessive amount of DNA would hinder electrophoresis in the capillaries, resulting in a shift of the peaks. This phenomenon may render the direct comparison of WDF and possible candidate varietal references difficult, especially in blended wines where more than a single variety contributes to the DNA admixture. The alleles composing the wine and grapevine respective genetic profiles derive from validation of observed alleles in both technical replicas (from 3 to 6, depending on the wine complexity) and multiple experiments. The SSR allele size is obtained using plant references as internal calibrators in each experiment and the interpretation of WDF and plant profiles takes into account the most recurrent values of allele size. Therefore, it is highly recommended to strictly follow an established protocol which includes the systematic use of technical replicas of wines and of plant references, and an accurate phase of data interpretation and genotyping assignment, when possible supervised by an external party skilled at genotyping.

### DNA testing and analytical traceability in wines and diagnostic markers

Concerning the choice of the marker type to be used in WDF, there are several options that can be undertaken. Currently, there are technical limitations for using SNPs for wine varietal diagnosis, especially related to the relatively high quantity of template DNA required for carrying out the analysis, which is not compatible with the average quantity of grapevine DNA extractable from wines. An interesting approach that permits by-passing PCR and labeling of probes consists in the use of bio-sensors [29]. This method is very sensitive and is promising for fast varietal determination, but the diagnostic capability is focused on specific sets of cultivars where the target genes have been re-sequenced in a restricted panel of varieties.

The use of SSR-based genotyping guarantees a universality trait, in the sense that any variety can be detected in a wine once the profile is known.

General issues on wine varietal identification pose a question regarding the integration of multiple analytical approaches (chemical profiling, molecular profiling). Even in molecular-based wine fingerprinting, the choice of marker type (SNPs, INDELs, SSRs) to be best used with wine remains an issue to be further discussed. It is feasible that molecular-based techniques for food and wine authenticity assessment will be extended and implemented in the light of new bioinformatics and genomics knowledge, applicable in the near future to the grapevine.

In a market characterized by growing competitiveness and ever-expanding borders it is necessary to inspire a positive interest in wine. Consumers are increasingly attracted and influenced by a number of attributes that affect product value, such as guarantees declared on labels like DOC and DOCG, product name, genuineness, and transparency. The phenomenon of counterfeiting and adulteration are dangerous to both producer reputation and human health. Technological analysis is a tool that allows producers to protect their wine and strengthens their relationship with consumers. For this reason, anti-counterfeiting technologies are increasing as effective and incontrovertible strategic tools for protecting wine lovers. However, in a globalized market analytical traceability is still only voluntarily adopted. Controls are not regularly undertaken on imported wines or on national products. Therefore, the systemic introduction of analytical methods for assessing wine genuineness before market delivery seems essential. It is likely that Governmental organizations’ deficient control systems will lead to wide-spread frauds on the worldwide wine market. Identifying the predominant variety in a blend helps to understand if the wine was produced according to local and national regulations.

### Perspective: Towards a decision support system for wine authentication

Here we would like to contextualise our work within a general framework for assessing a wine. In other words, our bioinformatics approach needs to be seen as part of a roadmap towards integrative (nucleic acids, metabolites, chemicals, cultural, etc.) wine properties. How to draw this roadmap?

The statistical methodologies inspired by bioinformatics used in this paper demonstrate how there are common tools shared between WDF elaborations and methods based on algorithms known for phylogenies reconstruction and statistical clustering. There are multiple bioinformatics and statistical analysis of data that may apply to the wine case. The Neighbor-Joining associated to statistical hierarchical clustering estimating the confidence intervals were the most suitable tools in our hands for depicting the varietal nature of the wines by WDF. It should also be noticed that the power of the analysis will be improved at the increasing of wine ontology and genetic databases.

Productive data utilization in wine quality assessment brings evidence-based inference into play. Consequently, systems-integrated data put the focus on methodological instruments to be considered in this work as Evidence Synthesis. Signals may be referred to patterns based on Multiparameter Evidence Synthesis. Models for potentially biased evidence in meta-analysis use empirically-based priors to build a coherent and flexible analytical framework that accommodates a disparate body of chemical and bioinformatics evidence available regarding admixture and prevalence estimation.

Data integration and evidence synthesis could be generated by using methods such as Decision trees and Random Forests. Decision trees are commonly used in disease classification and prediction of knowledge discovery in medical field [64]; it graphically illustrates its final output in a tree based model which does not require detailed explanation for data scientist or clinicians. It is widely used in modelling and prediction as it encompasses many advantages [65]. Moreover, RF classifier can be modelled for both continuous and categorical variables and also handle missing values.

Another meaningful way to combine chemical composition and bioinformatics is to use a multilayer network [66] which provides a meaningful description of both social, nutritional and metabolic networks [67]. Following [68, 69, 70, 71], a multilayer network M is a family of graphs that can be directed, undirected, weighted or unweighted and defines the layers of M (for example one layer could represent the nucleic acids information, another layer the chemicals); there is a set of interlayer connections between nodes of different layers.

Taking into account the previous assumptions and descriptions, the matrix M could be written as:

$$M\;=\;(\begin{array}{cccc} A_{1} & \omega_{12}Ι & \cdots \; & \omega_{1h}I\\ \omega_{21}Ι & A_{2} & \cdots & \omega_{2h}I\\ \vdots & \vdots & \ddots & \vdots \\ \omega_{h1}Ι & \omega_{h2}Ι & \cdots & A_{h} \end{array})$$

where multiplex network formed by h layers; A_1_,…,A_M_ are the adjacency matrices the ω_ij_ represent the interlayer interaction strength from layer i to j and I is the corresponding identity matrix dependencies (69). In a single-layered-network with unweighted edges a useful property is that the number of walks of length k between the nodes p and q is given by the p,q-entry of the kth power of the adjacency matrix of the network. In a multiplex network formed of unweighted graphs it follows that the walks of length k in the multiplex are given by entries of M_k_. Let’s now discuss how to model wine with multiplex networks. The strength of interaction between each data type can be modeled by a weight, connecting each layer in the multiplex.

In each layer, each node has a weighted, undirected edge connecting it to every other node in the same layer. In addition, each wine is connected to itself in every other layer by the strength of interaction between the data types. In this case, we consider that the strength of interaction is undirected and symmetric, i.e. ω_ij_ = ω_ji_. Since the weight between nodes is a measure of similarity or information shared between the nodes, it follows that the weight of the path provides a measure of information flowing through the path.

There are a number of ways we can provide a new measure of similarity between two nodes given the properties of the multiplex network. One way would be to take a mean of the direct paths connecting each wine to and from another wine in each and every layer.

**Fig 9:**
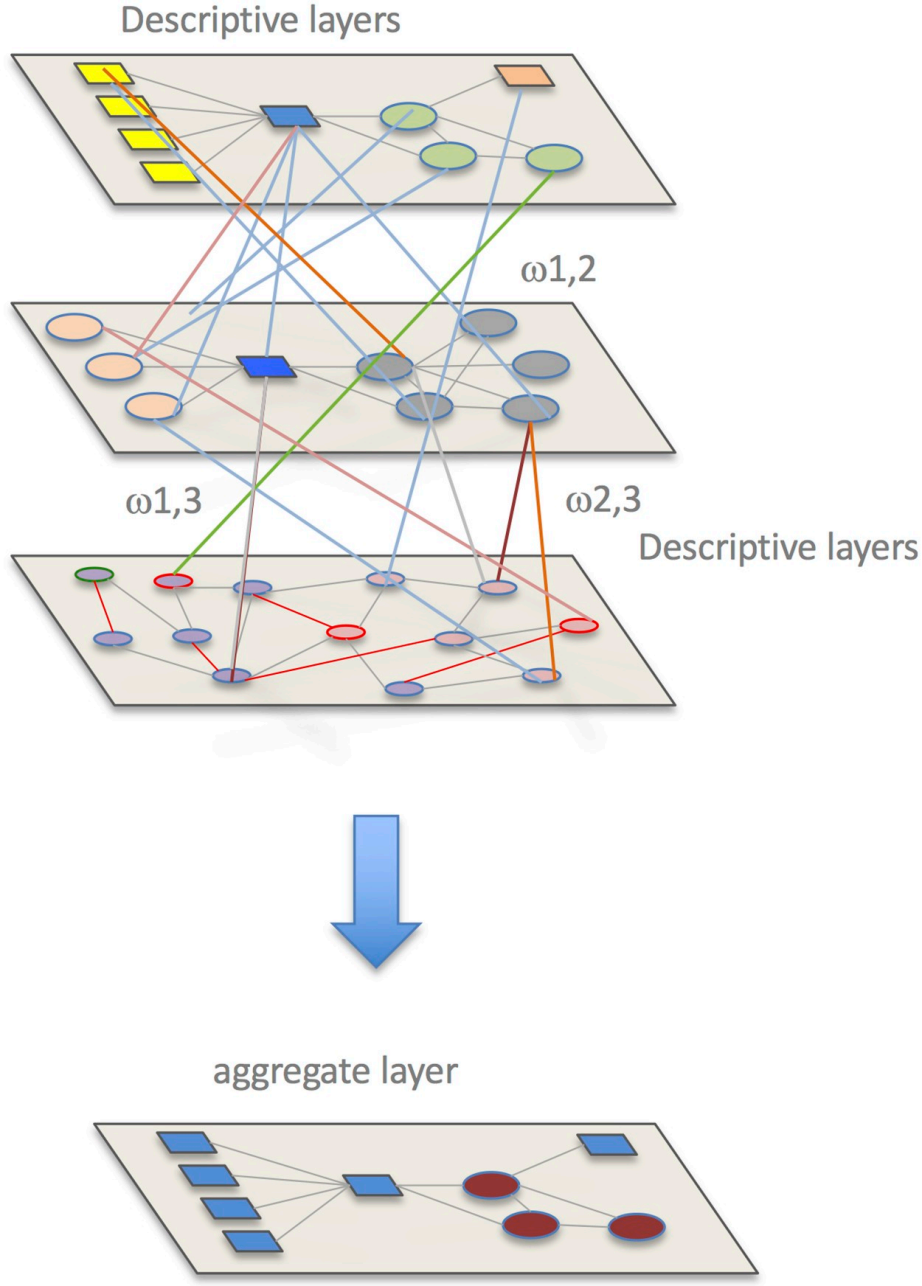
Method of aggregating different layers using regression or clustering approaches. Varying ω using the method of aggregating on unfiltered layers which represent biological, chemical, commercial, manifacturing, environmental, historical and other information.

In many situations, a pair of nodes in a network does not communicate only through the shortest-path routes connecting both nodes, but also through all possible routes connecting both nodes. The number of these possible routes can be enormous. Moreover, the information can also go back and forth before connecting the pair of nodes. In multiplex networks, a communicability metric between two nodes p and q, is a weighted sum of all walks from p to q (69). This leads to derive the relationships of the communicability between each pair of nodes. Finally, our model could be visualized in the following way as in Fig 9.

As previously discussed, the strength between layers in the multiplex, ω, represents a measure of dependency or strength of interaction between the layers. The edge weights between nodes represent a measure of similarity between nodes in the same layer, normalized between zero and one. Therefore, it is natural for the values of ω to represent a measure of dependence between zero and one, where zero and one indicate independence and total dependence between the layers, respectively. The values of ω are not known a priori, and therefore we can view them as parameters in our multiplex model. Clearly, if we vary the values of ω we should expect that the communicability between nodes in the multiplex will vary, and hence the aggregate layer is a function of all the ω values. We wish to use the multiplex model to predict the response of a new wine given knowledge of other wines. Therefore, given the data of a set of wines with known response we want our aggregate network to match the response network as closely as possible, i.e the difference between the edge weights in the aggregate and response network should be minimized. Aggregate clustering from all the layers could be considered. See [66] for further mathematical details.

### Wine forensics and wine age prediction

Given that the bioinformatics elaboration of WDFs complies with the need of verifying the varietal nature of a wine, it can be assumed that the DNA analysis carried out in the wine is somehow related to its chronological age; and together with multiple integrated input data, expresses information on the biological age of wine. Even if the chemical and physical status of DNA in wine is not well studied, one can suppose, based on what is described in other fields of study, that the average age of wines is not at risk of genetic information loss. In fact, it is reported by palaeogeneticist that DNA has a 521-year half-life and that it would cease to be readable after roughly 1.5 million years [59]. In spite of the developments made in wine DNA profiling some may believe that grapevine DNA recovered in an aqueous phase, such as wine, will have no forensic value; and therefore argue against the reliability of DNA analysis for wine varietal ascertainment. Recent forensic work demonstrates how the latent DNA fingerprint can be recovered even from saline seawater, and that the reliability of the test depends very much on specific environmental conditions [72]. It is reasonable to suppose that in the alcoholic wine environment total DNA, including the minority deriving from the grapevine, may be able to survive at least several years. It is therefore not to be excluded that DNA analysis will comply with legal issues of wine composition in the future, and in special cases (e.g. historical valuable wines) will act as an indirect index to estimate wine age.

### Bioinformatics, WDF and synthetic wines

In agreement with increasing sensitiveness toward the natural equilibrium of planet and ecosystem preservation, and in light of embracing a cruelty-free philosophy, lab-grown food is progressively expanding in research centers and a near, future market [73]. Startups around the world aim at ambitious goals, including the production of meat without animals (Memphis Meat, San Francisco, CA, USA), *in vitro* produced fish fillets (Finless Food, San Francisco, CA, USA), nutritionally optimized mayonnaise, yogurt, cheese without animal ingredients (NotCo, Santiago de Chile, Chile) and fine "synthetic" wines with no grapes (Ava winery, San Francisco, CA, USA). In the future the development of integrated and advanced bioinformatics and molecular analytical tools may render immediate detection of natural wines against "synthetic" ones based on DNA detection of DNA residue in the bottle. In addition, thanks to the wide genomic knowledge of the *Vitis* genus family members, the wine molecular recognition databanks will progressively expand, allowing for the classification of large sets of wine types.

## Conclusions

**Fig 10:**
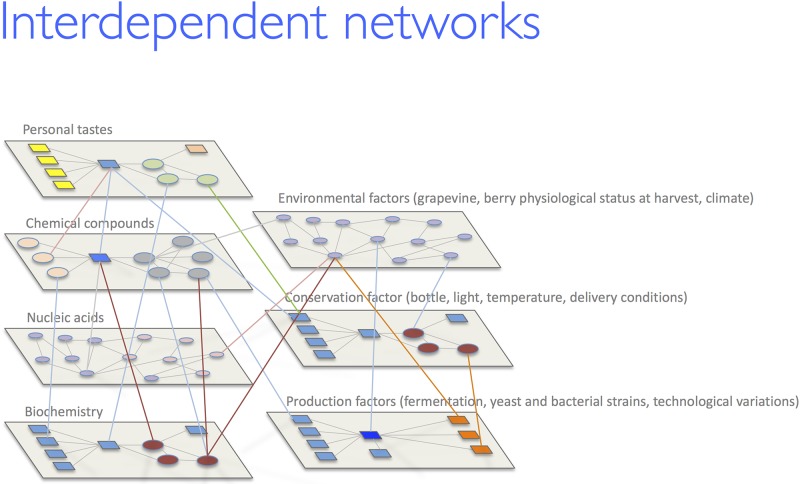
Interdependent informative inputs and data networking for wine ontology implementation. Multidimensional networking among the main features influencing a wine. Wine traits (DNA profile, metabolomic and chemical profiling) are in turn interconnected to personal tastes and general environmental factors (e.g. climate). Bioinformatics elaborates multilayer input data contributing to wine ontology fed by semantic web languages.

**Fig 11:**
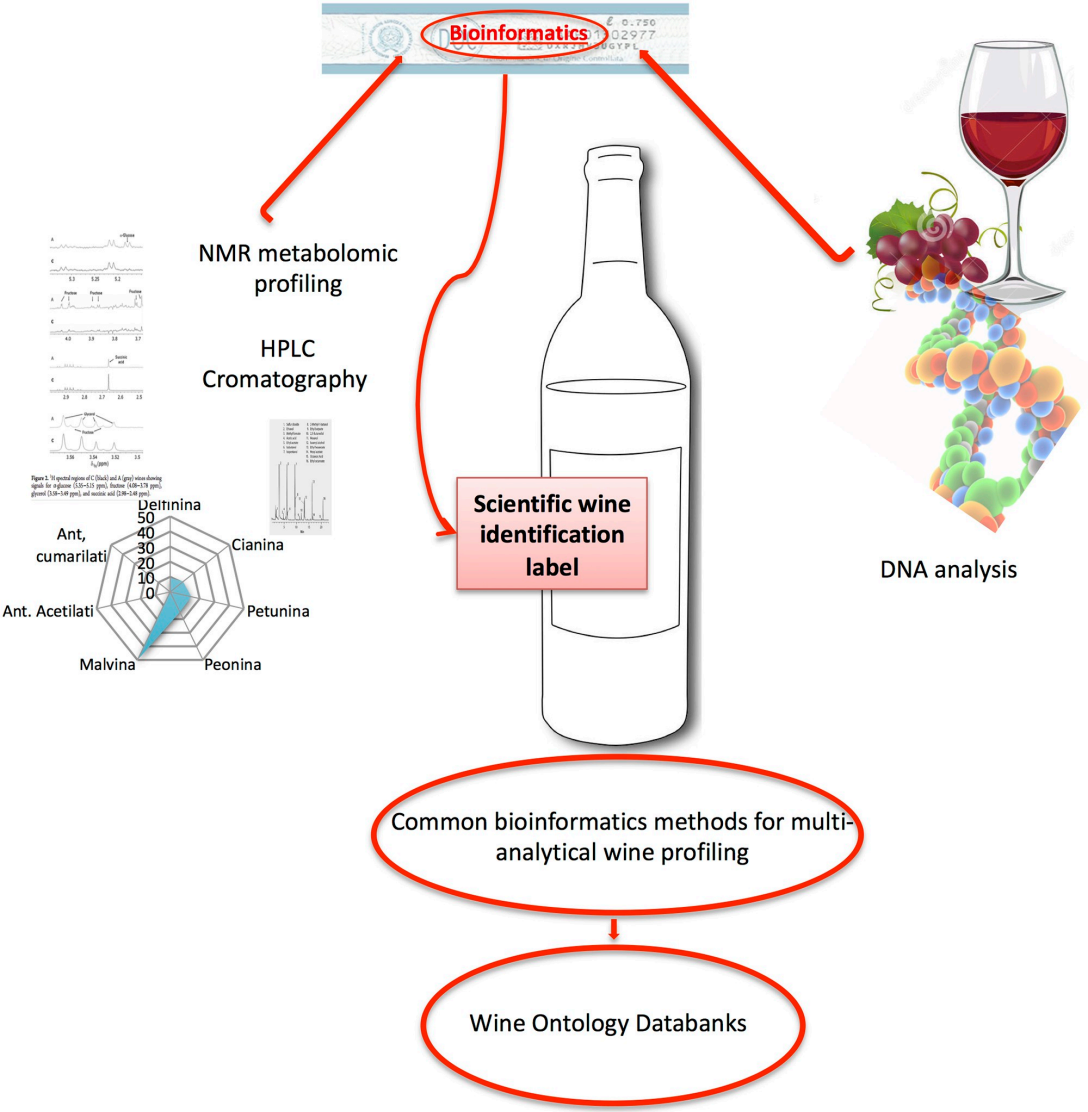
Towards a new wine ontology databank system. According to a new wine ontology model based on a bioinformatics elaboration of multidimensional wine traits, a scientific unambiguous labeling of wine can be obtained. Bioinformatics is a common, essential tool for the validation of multiple, analytical approaches to wine authentication: molecular, chemical, metabolomic profiling, which merge into a comprehensive wine ontology databank fed by bioinformatic tools.

The bioinformatics applied to DNA analysis in wine reveals to be an effective tool, confirming the reliability of DNA analysis for wine varietal assessment that must comply to legal requisites concerning wine production and commercialization. Data presented here can be implemented by multidisciplinary inputs (Fig 10) deriving from progresses in the molecular, genomics, metabolomics, historical and cultural fields concerning the wine world, leading to future integration of portals existing on the web dedicated the wine ontology [74]. Bioinformatics is a common, essential tool for the validation of multiple, analytical approaches to wine authentication. Molecular, chemical, and metabolomic profiling merge into a comprehensive wine ontology databank fed by bioinformatics tools [75] (Fig 11). The research field is growing of importance and expanding in several directions/dimensions. We believe that the discussion of the results should be coupled with well grounded considerations of how dry-lab methodologies could transform the field towards elucidation of actionable target markers. Our vision is that the biomolecular analysis applied to wine varietal diagnosis can be seen as the first proof of a wider wine quality concept; indexing the biological age of wine which is the result of multiple complex biotic and abiotic molecular interactions, more than mere physical timing measured in years.

## Supporting information

S1 Fig SSR profiles of several wine types. Electropherograms of commercial and experimental wines show alleles correspondence between wine and respective reference grapevines.
(PDF)

S1 Table Example of WDF for monovarietal wines. Sangiovese SSR allelic profiles merged into a single data set.
(PDF)

S2 Table Quantity and quality of the extracted DNA from wines. Total DNA concentration was estimated using the NanoDropTM 1000 spectrophotometer (Thermo Fisher Scientific). V. vinifera DNA concentration was obtained using a TaqMan probe targeting the endogenous gene VvNCED2 in a RT-PCR assay as described in Bigliazzi et al. 2012.
(PDF)

S3 Table Example of WDF for commercial wines available in the US market. Varietal white wines SSR allelic profiles merged into a single data set.
(PDF)

S4 Table Example of WDF for commercial wines available in the US market. Varietal red wines SSR allelic profiles merged into a single data set.
(PDF)
