# Supplementary material for: How to integrate wet lab and bioinformatics procedures for wine DNA admixture analysis and compositional profiling: Case studies and perspectives
Source: PLoS One. 2019 Feb 12;14(2):e0211962. doi: 10.1371/journal.pone.0211962 (PMC6376920; doi:10.1371/journal.pone.0211962)
Supplement: S1 Fig — Electropherograms of commercial and experimental wines show alleles correspondence between wine and respective reference grapevines. (PDF) [file pone.0211962.s001.pdf]

SSR profile comparison between Brunello di Montalcino, DOCG monovarietal wine and grapevine

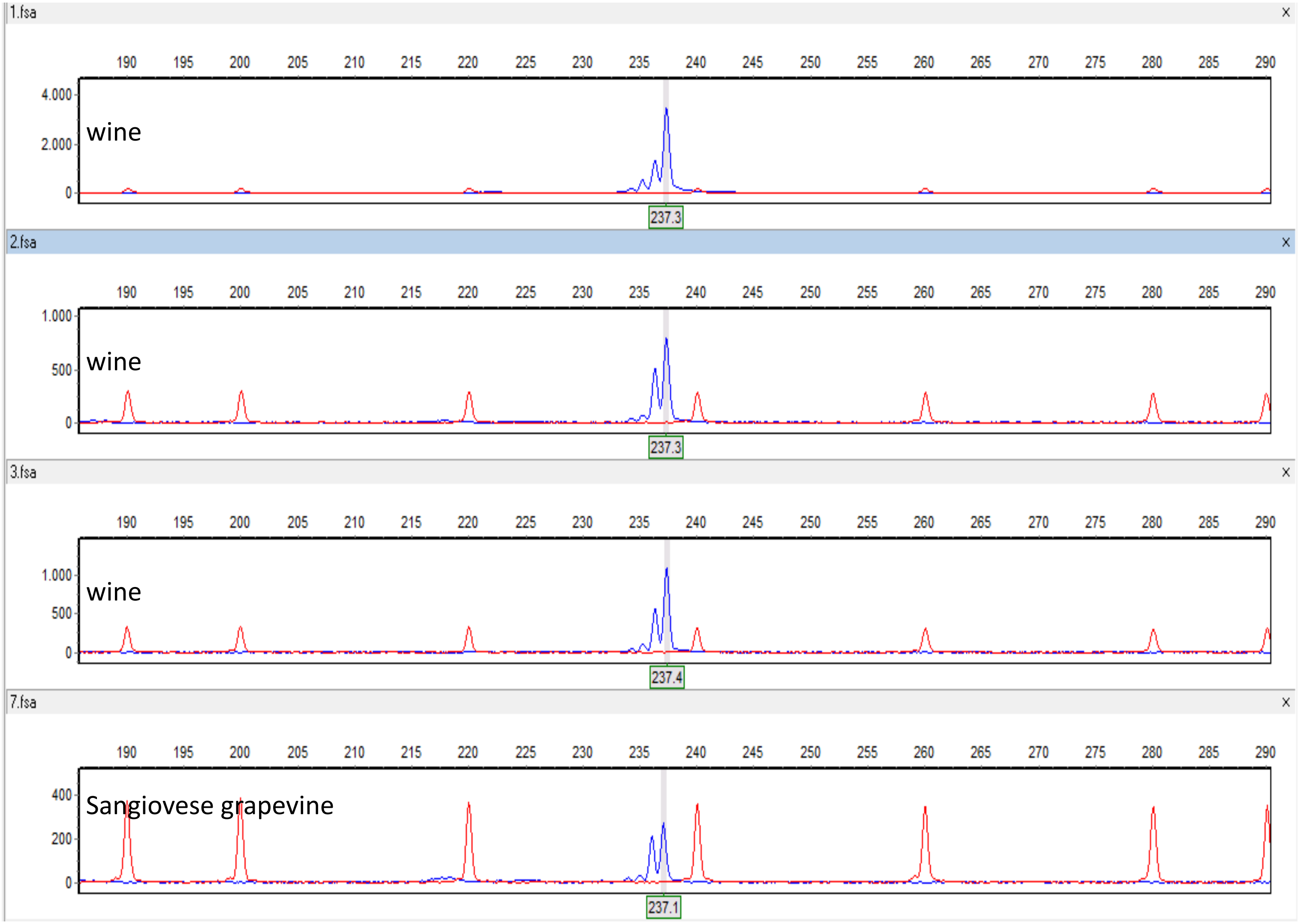

Electropherogram of the Brunello di Montalcino DOCG monovarietal wine analysed by Capillary Electrophoresys (CE) at VVMD34.

SSR profile comparison between Brunello di Montalcino, DOCG monovarietal wine and grapevine

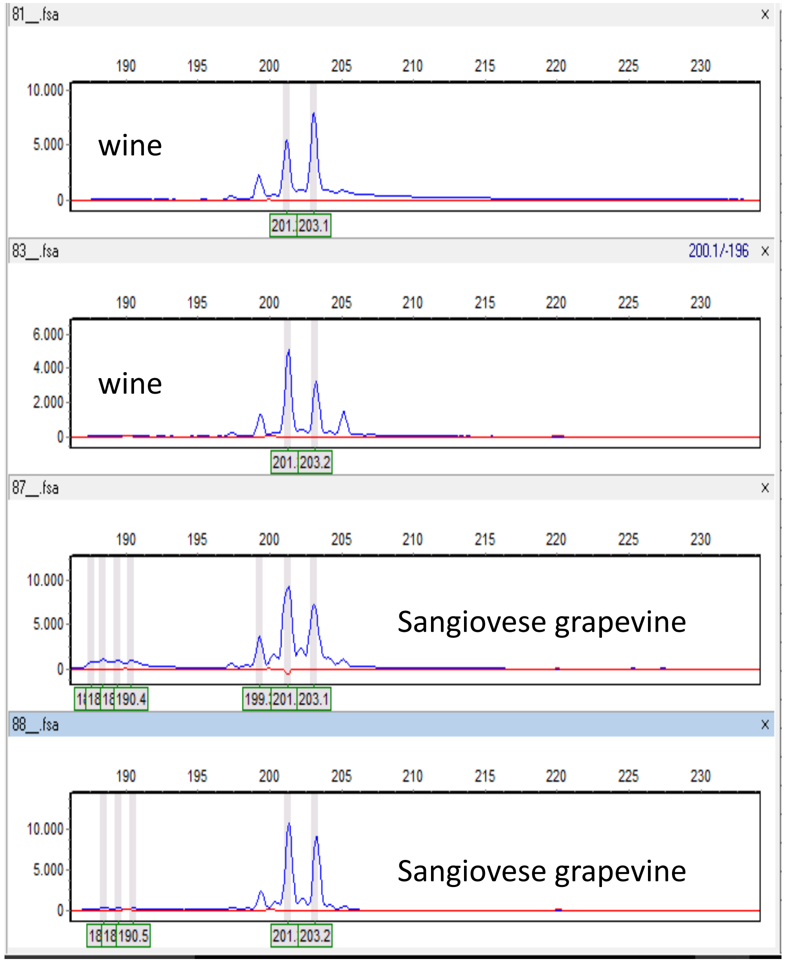

Electropherogram of the Brunello di Montalcino DOCG monovarietal wine analysed by Capillary Electrophoresys (CE) at VrZag21.

SSR profile comparison between IN7 monovarietal experimental wine and grapevines

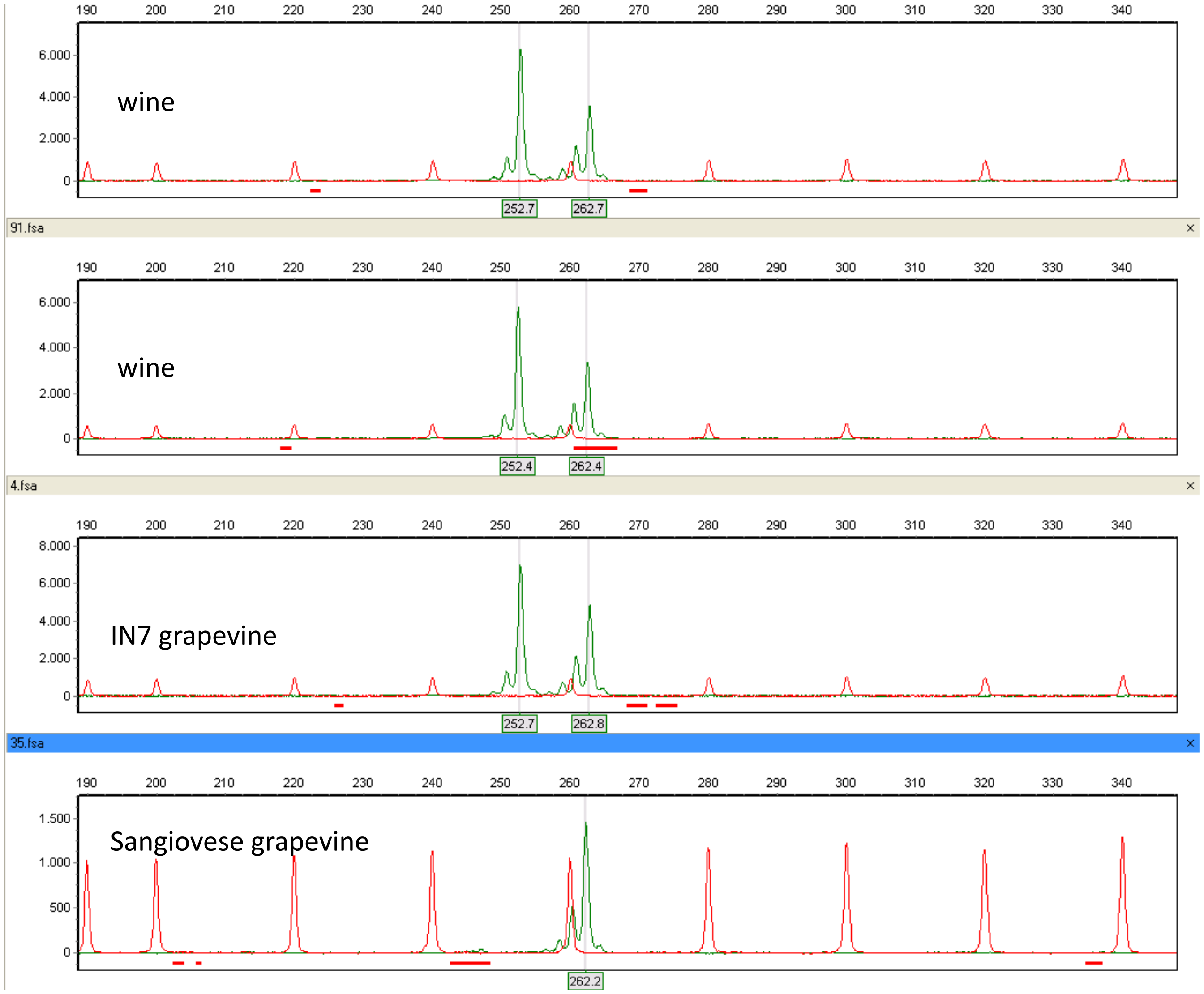

Electropherogram of the IN7 monovarietal, experimental wine analysed by Capillary Electrophoresys (CE) at VVMD36.

SSR profile comparison between Valpolicella Classico, DOC blended wine and grapevines

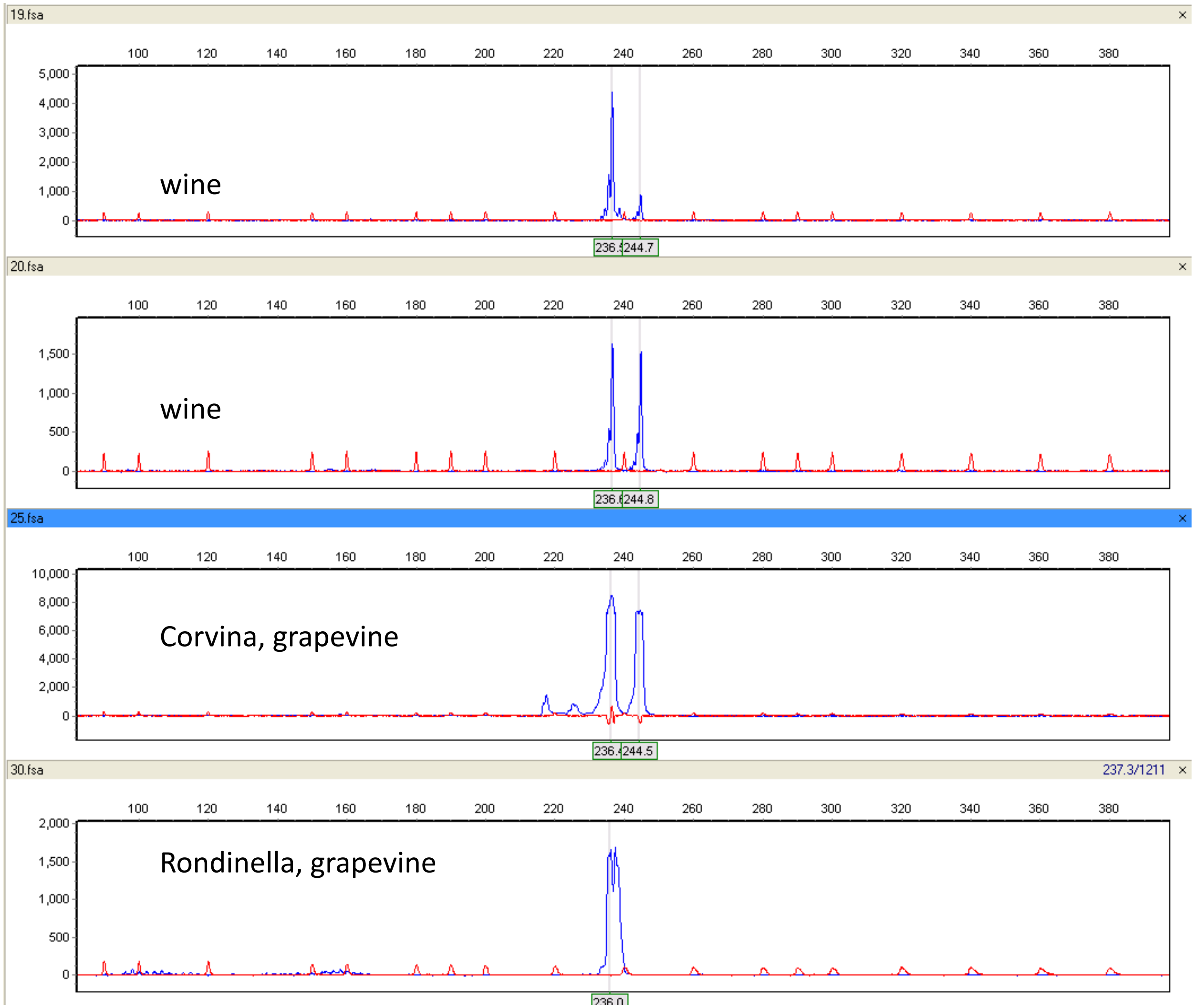

Electropherogram of the Valpolicella classico DOC, blended wine analysed by Capillary Electrophoresys (CE) at VVMD34.

SSR profile comparison between unknown red varietal wine 947 and grapevines

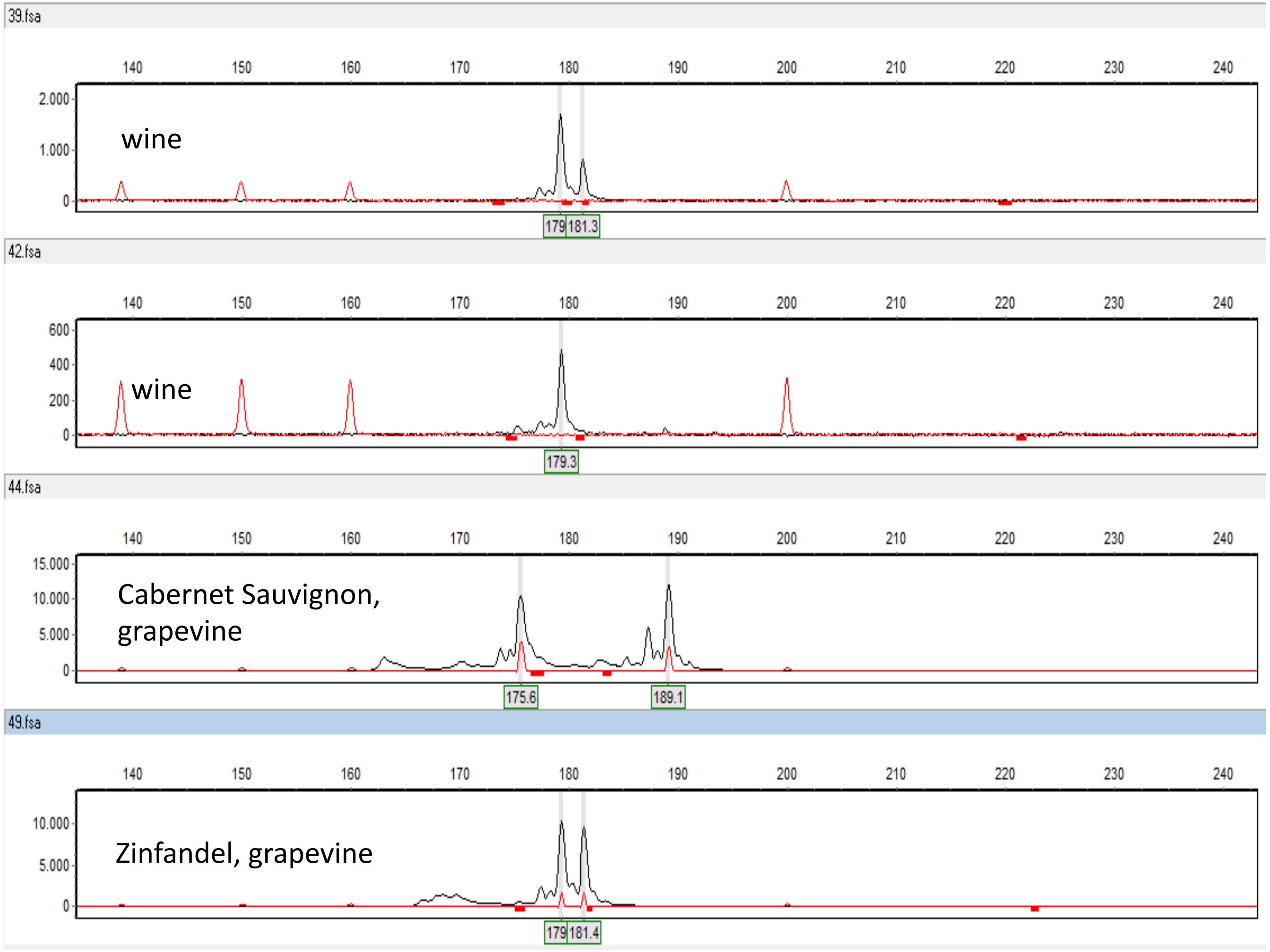

Electropherogram of the unknown varietal wine US 947 analysed by CE at SSR loci VVMD27.

SSR profile comparison between unknown red varietal wine 947 and grapevines

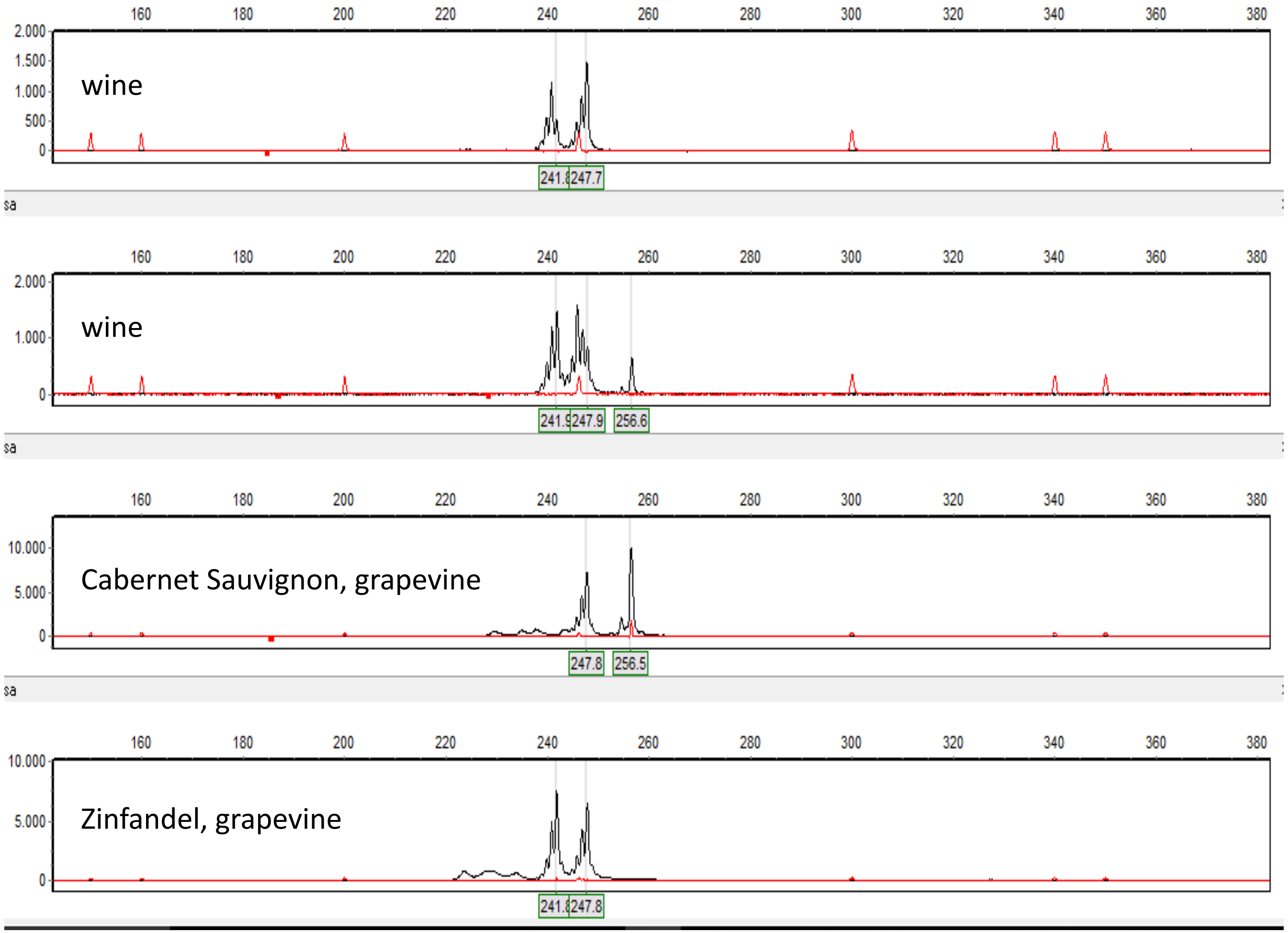

Electropherogram of the unknown varietal wine US 947 analysed by CE at SSR loci VVMD21.

SSR profile comparison between unknown red varietal wine 947 and grapevines

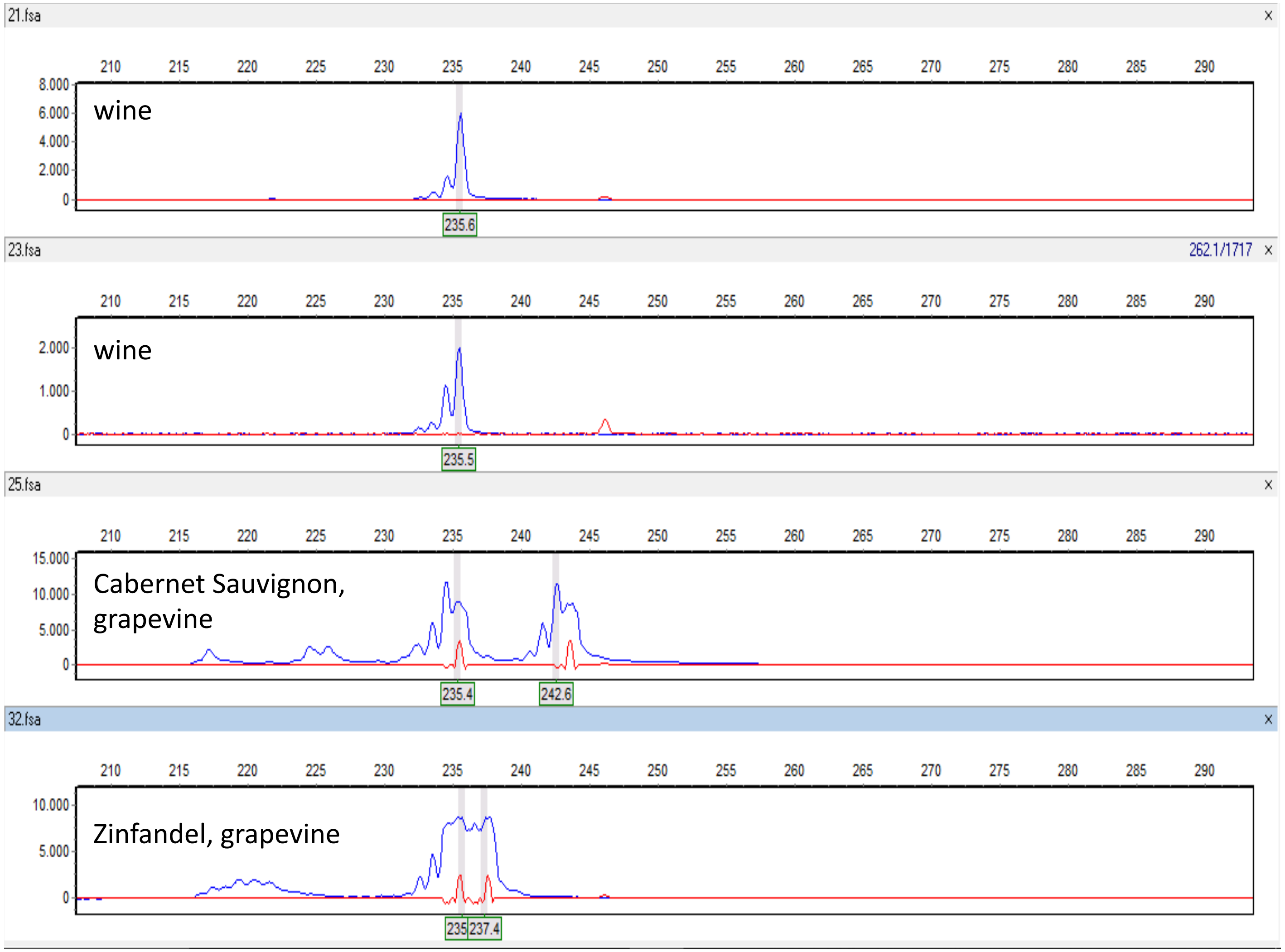

Electropherogram of the unknown varietal wine US 947 analysed by CE at SSR loci VVMD34.

SSR profile comparison between unknown red varietal wine 947 and grapevines

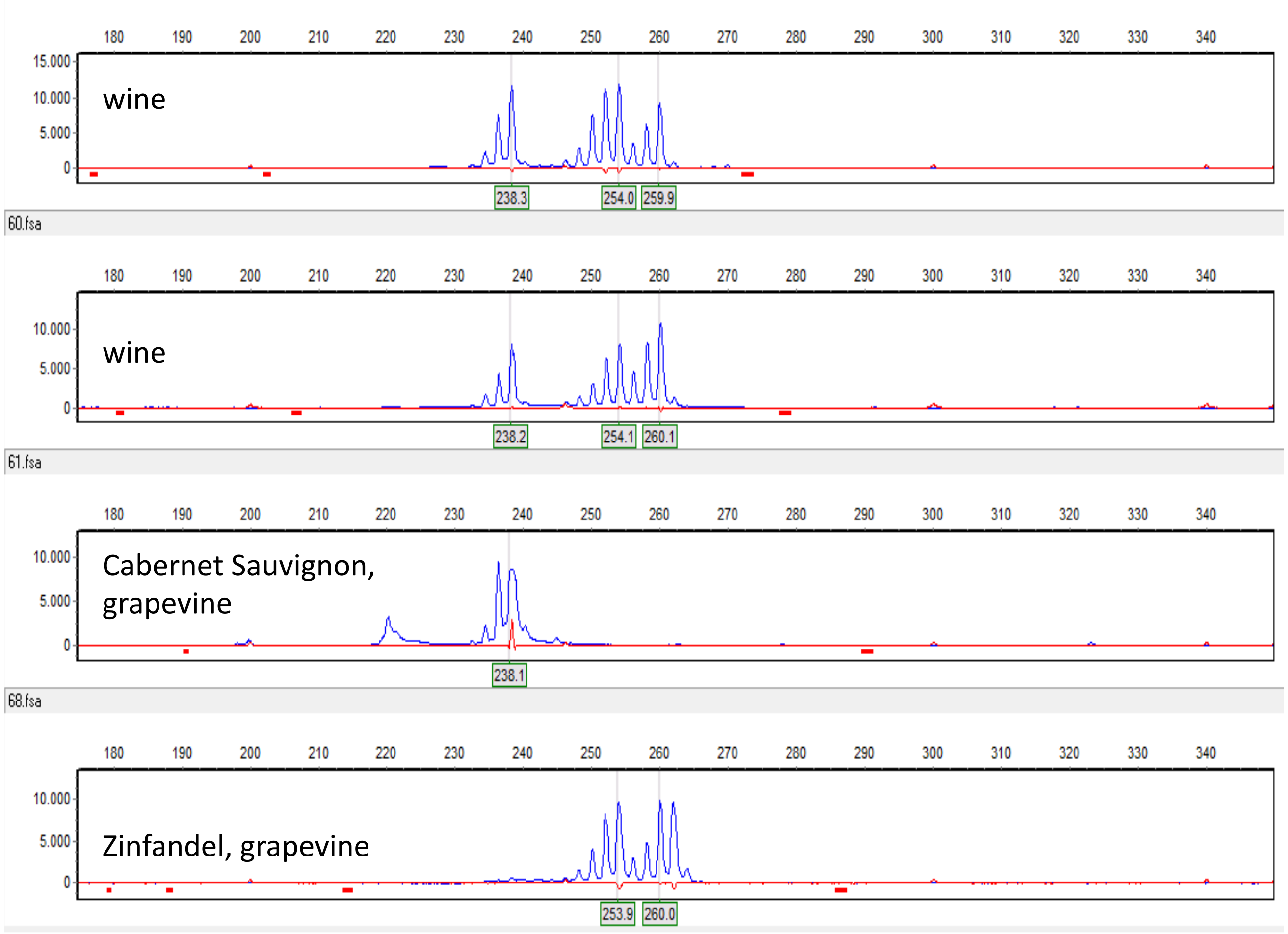

Electropherogram of the unknown varietal wine US 947 analysed by CE at SSR loci VVMD32.
