# Supplementary material for: How to integrate wet lab and bioinformatics procedures for wine DNA admixture analysis and compositional profiling: Case studies and perspectives
Source: PLoS One. 2019 Feb 12;14(2):e0211962. doi: 10.1371/journal.pone.0211962 (PMC6376920; doi:10.1371/journal.pone.0211962)
Supplement: S1 Table — Sangiovese SSR allelic profiles merged into a single data set. (PDF) [file pone.0211962.s002.pdf]

| SSR marker | | VVMD24 | VVMD34 | VVMD27 | VVMD21 | VVMD25 | VrZag83 | VVMD32 |
|---|---|---|---|---|---|---|---|---|
| **wines** | | | | | | | | |
| | | | | | | | | |
| **Brunello di Montalcino Caprili 2014** | | 208-214 | | 179-185-189 | 243-249 | 240-248-254 | | 253-257 |
| **Rosso di Montalcino Az. Palazzo 2013** | | 208-214 | | 179-185-189 | 243-249 | 240-248 | | 253-257 |
| **Small scale fermented wine CB17** | | 208-214 | | 185-189 | 249 | | | |
| **Small scale fermented IN7** | | 212-216 | | 179-185-189 | 243 | | | 253-263 |
| | | | | | | | | |
| **grapevines** | | | | | | | | |
| | | | | | | | | |
| | | | | | | | | |
| **Sangiovese** | | 208-214 | 238 | 179-185 | 243-249 | 240 | 191-196 | 253-257 |
| **Cabernet S.** | | 208 - 216 | 238-246 | 176-189 | 249-258 | 240-249 | 202 | 240 |
| **Merlot** | | 208-212 | 238 | 189 | 243-249 | 240-249 | 196-202 | 240 |
| **Pinot Nero** | | 214 -216 | 238 | 186-189 | 249 | 240-249 | 190-202 | 240-271 |
| **Zinfandel** | | 208 | 238-246 | 180-182 | 243-249 | 240 | 190-196 | 255-263 |
| **Sangiovese variant Caprili** | | 214-217 | 238-240 | 179-185 | 249 | 240-254 | 189-191 | 253-271 |
| **Sangiovese variant Case Basse CB17** | | 208-214 | | 185-189 | 249 | 240-254 | 191-201 | |
| **Sangiovese variant Case Basse IN7** | | 212-216 | | 179-185 | 233-243 | 237-240 | 191-201 | 253-263 |

Each allele was scored in a mtarix as present (1) or absent (0) in each individual in the population.
