# Supplementary material for: How to integrate wet lab and bioinformatics procedures for wine DNA admixture analysis and compositional profiling: Case studies and perspectives
Source: PLoS One. 2019 Feb 12;14(2):e0211962. doi: 10.1371/journal.pone.0211962 (PMC6376920; doi:10.1371/journal.pone.0211962)
Supplement: S2 Table — Total DNA concentration was estimated using the NanoDropTM 1000 spectrophotometer (Thermo Fisher Scientific). V. vinifera DNA concentration was obtained using a TaqMan probe targeting the endogenous gene VvNCED2 in a RT-PCR assay as described in Bigliazzi et al. 2012. (PDF) [file pone.0211962.s003.pdf]

Wine name	Total DNA (ng/µL)	260/280 ratio	260/230 ratio	*V. vinifera* NCED2 (ng/mL)
1. IN7	131.15	1.39	0.38	196.5
2. CB17	95.15	1.10	0.36	161.5
3. Brunello di Montalcino	29.44	1.48	0.24	45
4. Rosso di Montalcino	35.27	1.68	0.41	38
5. Alicante	42.78	1.25	0.23	20.3
6. Cabernet Sauvignon, varietal	83.74	1.28	0.33	31.5
7. Valpolicella Classico	29.84	1.82	0.45	37.5
8. Merlot, varietal	32.15	1.57	0.48	27
9. Amarone	27.44	1.43	0.79	4.1
10. Vernaccia di San Gimignano 1	12.14	1.67	0.55	19.8
11. Vernaccia di San Gimignano 2	15.17	1.42	0.61	11.2
12. Unknown red varietal wine 947	38.12	1.15	0.32	29
13. Unknown red varietal wine 949	41.20	1.53	0.36	30.7
14. Unknown red varietal wine 950	32.46	1.65	0.18	29.1
15. Unknown red varietal wine 951	39.7	1.72	0.43	18.8
16. Unknown white varietal wine 940	27.8	1.8	0.37	28
17. Unknown white varietal wine 948	26.5	1.63	0.50	21
18. Unknown white varietal wine 953	31.2	1.7	0.32	34

Quantity and quality of the extracted DNA from wines. Total DNA concentration was estimated using the NanoDropTM 1000 spectrophotometer (Thermo Fisher Scientific).
V. vinifera DNA concentration was obtained using a TaqMan probe targeting the endogenous gene VvNCED2 in a RT-PCR assay as described in Bigliazzi et al. 2012.
