# Supplementary material for: How to integrate wet lab and bioinformatics procedures for wine DNA admixture analysis and compositional profiling: Case studies and perspectives
Source: PLoS One. 2019 Feb 12;14(2):e0211962. doi: 10.1371/journal.pone.0211962 (PMC6376920; doi:10.1371/journal.pone.0211962)
Supplement: S3 Table — Varietal white wines SSR allelic profiles merged into a single data set. (PDF) [file pone.0211962.s004.pdf]

SSR marker	VVMD21	VVMD25	VRZAG83	VVMD24	VRZAG21	VVMD27	VVDM32	VRZAG64	VVMD34
**wines**									
**wine TTB 953**	243-249	238-240-260	190-200	206-209-214-216	199-201-203	180-188	239-251	137-139	
**wine TTB 948**	243-245-249	236-240-248-252	186-188-190-194-200	206	199-201-203	176-178-182-184-188-193	239-251-270	137-139-143-159	235-237
**wine TTB 940**	243-249	238-240-248-254		206-214	199-201-203-205	178-180-182-184	237-239-253-260-270	153-163	237
**grapevines**									
**Riesling grapevine**	249	248-254	188-194	206-214	201-205	180-188	251-270	137-159	237
**Sauvignon Blanc grapevine**	243-249	240-248	190-200	214	203-205	174-188	239-255	139-143	237-245
**Chardonnay grapevine**	249	238-254	188-200	206-214	199-205	180-188	239-270	159-163	237

Each allele was scored in a mtarix as present (1) or absent (0) in each individual in the population.
