# Supplementary material for: How to integrate wet lab and bioinformatics procedures for wine DNA admixture analysis and compositional profiling: Case studies and perspectives
Source: PLoS One. 2019 Feb 12;14(2):e0211962. doi: 10.1371/journal.pone.0211962 (PMC6376920; doi:10.1371/journal.pone.0211962)
Supplement: S4 Table — Varietal red wines SSR allelic profiles merged into a single data set. (PDF) [file pone.0211962.s005.pdf]

SSR marker	VVMD24	VVMD34	VVMD27	VVMD21	VVMD25	VrZag83	VrZag21
**wines**							
**wine TTB-950**	208-213-214-216	238-242	184-186	243-249-258	240-241-243	190-193	198-200-202-206
**wine TTB-947**	208-211-213-215-216	237-246	175-180-183-186-189	243-249-257	240-242-249	190	200-206
**wine TTB-951**	208-213-216	236-238-241-246	180-184-186-189	243-249-259	240-249	193-196-203	200-202-204-206
**wine TTB-949**	208-212-213-214-216	238-242-246	176-180-186-189	249-258	240	192-194-196-202	200-202-204-206-210-223
**grapevines**							
**Cabernet S.**	208-216	238-246	176-189	249-258	240-249	202	200-206
**Merlot**	208-212	238	189	243-249	240-249	196-202	200
**Pinot Nero**	214-216	238	186-189	249	240-249	190-202	200-206
**Zinfandel**	208	237-239	180-182	243-249	240	190-196	200-206

Each allele was scored in a matrix as present (1) or absent (0) in each individual in the population.
